# Directional changes in Levallois core technologies between Eastern Africa, Arabia, and the Levant during MIS 5

**DOI:** 10.1038/s41598-021-90744-z

**Published:** 2021-06-01

**Authors:** James Blinkhorn, Huw S. Groucutt, Eleanor M. L. Scerri, Michael D. Petraglia, Simon Blockley

**Affiliations:** 1 Pan African Evolution Research Group, Max Planck Institute for the Science of Human History, Jena, Germany; 2 Centre for Quaternary Research, Department of Geography, Royal Holloway, University of London, Egham, UK; 3 Extreme Events Research Group, Max Planck Institutes for Chemical Ecology, The Science of Human History, and Biogeochemistry, Hans‑Knöll‑Strasse 8, 07745 Jena, Germany; 4 Department of Archaeology, Max Planck Institute for the Science of Human History, Kahlaische Strasse 10, 07745 Jena, Germany; 5 Institute of Prehistoric Archaeology, University of Cologne, 50931 Cologne, Germany; 6 Department of Classics and Archaeology, University of Malta, Msida, MSD 2080 Malta; 7 Human Origins Program, Smithsonian Institution, Washington, DC 20560 USA; 8 School of Social Science, The University of Queensland, Brisbane, QLD 4072 Australia; 9 Australian Research Centre for Human Evolution (ARCHE), Griffith University, Brisbane, Australia

**Keywords:** Archaeology, Archaeology

## Abstract

Marine Isotope Stage (MIS) 5, ~ 130 to 71 thousand years ago, was a key period for the geographic expansion of *Homo sapiens*, including engagement with new landscapes within Africa and dispersal into Asia. Occupation of the Levant by *Homo sapiens* in MIS 5 is well established, while recent research has documented complementary evidence in Arabia. Here, we undertake the first detailed comparison of Levallois core technology from eastern Africa, Arabia, and the Levant during MIS 5, including multiple sites associated with *Homo sapiens* fossils. We employ quantitative comparisons of individual artefacts that provides a detailed appraisal of Levallois reduction activity in MIS 5, thereby enabling assessment of intra- and inter-assemblage variability for the first time. Our results demonstrate a pattern of geographically structured variability embedded within a shared focus on centripetal Levallois reduction schemes and overlapping core morphologies. We reveal directional changes in core shaping and flake production from eastern Africa to Arabia and the Levant that are independent of differences in geographic or environmental parameters. These results are consistent with a common cultural inheritance between these regions, potentially stemming from a shared late Middle Pleistocene source in eastern Africa.

## Introduction

*Homo sapiens* are first identified in Africa ca. 300 thousand years ago (ka)^1^, emerging in a mosaic of sub-structured populations across the continent^2^. The earliest appearance of *Homo sapiens* in Eurasia is consistently being extended deeper in time, and though data remains sparse, current fossil and archaeological evidence may indicate a late Middle Pleistocene expansion of *Homo sapiens*^3–5^. Although often considered to represent a ‘failed’ and geographically restricted dispersal, the recovery of *Homo sapiens* fossils in the Levant during Marine Isotope Stage (MIS) 5 (130-71 ka) at the sites of Skhul^6,7^ and Qafzeh^8^ is increasingly supported by evidence from elsewhere in Asia to indicate a substantive population expansion at this time^9^. Among multiple potential waves of expansions out of Africa, dispersal during MIS 5 appears to represent a step change in terms of scale, certainly with regards to its manifestation in the fossil and archaeological records^10^. Evidence to support a widespread expansion of *Homo sapiens* in MIS 5 has been identified from southern and eastern Asia^11^. However, the past decade has seen significant enhancement of the Palaeolithic record in Arabia, including the recovery of a *Homo sapiens* fossil in association with Middle Palaeolithic finds at ca. 90 ka^12^ and human footprints dating to 120 ka^13^. Critically, this enhances the possibility for detailed inter-regional examination of MIS 5 dispersals of *Homo sapiens *at the interface of Africa and Eurasia.

The geographic significance of Southwest Asia is extended by evidence that *Homo sapiens* and Neanderthals likely interbred shortly after the former expanded from Africa^14^. Yet, the majority of research undertaken in the Levant is concentrated in cave sites from Israel and Lebanon, which are ecologically and geographically atypical of wider southwestern Asian landscapes^15^. The growing body of evidence emerging from research in Arabia offers an important opportunity to examine how expanding populations of *Homo sapiens* engaged with the diversity of landscapes encountered immediately beyond the Africa-Eurasia threshold^12,16,17^. Although dated fossil specimens of *Homo sapiens* are critical to establish the tempo of demographic change in southwest Asia, they remain sparsely distributed and identifying their presence is dependent upon both preservation conditions and recovery through excavation of suitably stratified deposits. In contrast, stone tool assemblages that comprise the majority of the archaeological record are enduring, widespread, and numerous and can be informative about the timing and distribution of hominin occupations and the correspondence between stone toolkits and ecosystem variability. Stone tool technologies preserved in the Middle Palaeolithic records of Southwest Asia, are of course, influenced by raw material differences and alternate mobility strategies, but also learned behaviours, and accentuated vertical cultural transmission in small hunter-gatherer groups^18^. Examining patterns of variability within and between stone tool assemblages provides a critical means to explore cultural evolution, offering alternative insights into human evolution to those solely rooted in the fossil record.

**Figure 1:**
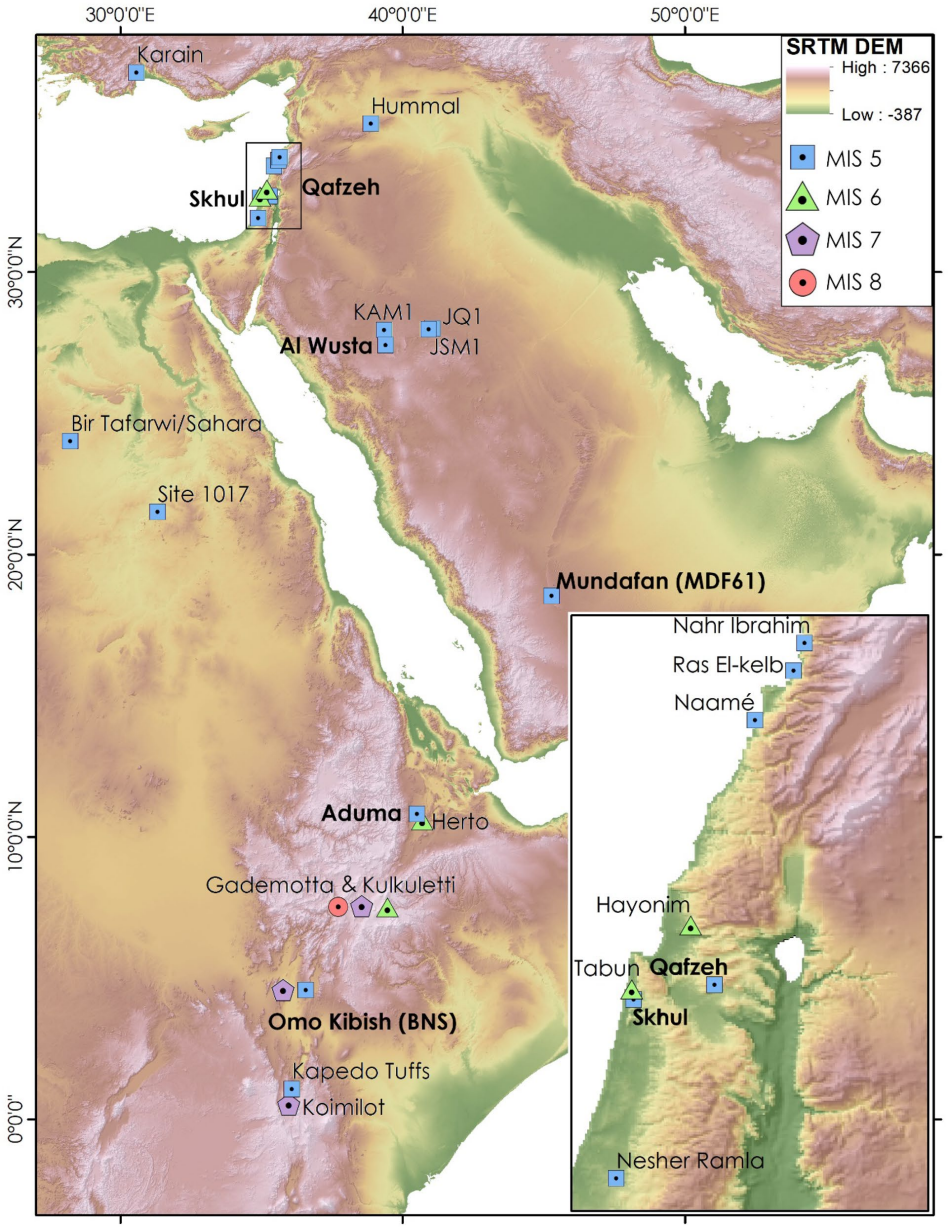
Map illustrating the distribution of sites studied here (named in bold) among sites with a prominent use of centripetal Levallois reduction (following Prévost and Zaidner^20^). A concentration of assemblages dating between MIS 8–6 is identified in eastern Africa, followed by more widespread appearance across Eastern/North-East Africa and Southwest Asia in MIS 5. Inset is a closeup of the central Levant. Data: SRTM (NASA)^25^. Figure produced using ArcMap 10.5 and GIMP 2.10.24.

Centripetal Levallois technology is a key component of stone tool production strategies associated with the expansion of *Homo sapiens* during MIS 5^10,19,20^ (Fig. 1). Centripetal Levallois reduction schemes are directly associated with *Homo sapiens* fossils at the sites of Skhul^21,22^ and Qafzeh^8^ in the central Levant, as well as at Al Wusta in northern Arabia^12^. This contrasts with the focus on unidirectional and convergent reduction schemes that predominate in later Middle Palaeolithic Levantine assemblages dating from MIS 4 (71–59 ka) onwards, frequently associated with Neanderthal fossils such as at Kebara^23^. This division in the dominant Levallois approach in assemblages is widely observed in the archaeological record in the Levant and is hinted at by the emerging Arabian record^24^. However, the extent to which variability exists within such chronologically and technologically distinct assemblages is not clear, highlighting the need to examine the scale of within and between assemblage variability. Assemblage-scale evidence suggests comparability in the focus on centripetal Levallois among MIS 5 assemblages between eastern Africa, Arabia and the Levant^20^, but no artefact-scale comparisons have been conducted to test this in detail.

Here, we quantitatively examine Levallois core attribute datasets from six sites (Table 1; Fig. 1). The sites date to MIS 5 and span across the interface of Africa and Eurasia. Three of the four sites are located in Southwest Asia; Qafzeh, Skhul and Al Wusta directly associate with *Homo sapiens* fossils. Cores assist in the assessment of patterns of cultural transmission as they represent residual forms of reduction processes, discarded after suitable flakes have been produced. Cores therefore contrast with retouched artefacts as the latter are subject to greater functional demands, resulting in potentially considerable transformation through their use life^26^. Similarly, flakes represent single removals from a reduction system, whereas cores can preserve features of a reduction system beyond a single removal. Patterns of reduction intensity are a critical factor in the examination of core assemblages^27^, and particularly the potential for significant changes in reduction approach and morphology as core volumes change. By examining cores that preserve all identifiable features of Levallois reduction schemes^28^, the impact of reduction intensity on patterns of morphological variability can be constrained. In particular, in order to identify a Levallois core, key elements of the design and preparation features must be evident, rather than further ad hoc flaking of cores beyond the application of a Levallois reduction sequence. Elsewhere, we have demonstrated considerable overlaps in Levallois core morphologies^29,30^ that likely reflect such design constraints, though substantive differences between flake and point production schemes are apparent^29^. Here, we focus on examining patterns of variability within Levallois flake cores. The current study is in accordance with research that examines variability in lithic attribute datasets across different domains of reduction activity within a quantitative framework, demonstrably resolving patterns of reduction behaviour at scales ranging from individual reduction sequences^31^ to inter-regional assemblage comparisons^32^.

**Table 1:** Archaeological sites, assemblages, and ages used in this study.

Region	Site	Assemblages	Age (ka)	Total cores studied	Key References
Eastern Africa	Aduma	A5	~ 80	76	^33^
	Omo Kibish	BNS	~ 104 ± 1	22	^34^
Arabia	Mundafan	MDF61	77.1 ± 8 to 95.6 ± 5.9	82	^35^
	Al Wusta		~ 85	42	^12^
Levant	Skhul		102 ± 26 to 119 ± 18	35	^7,36^
	Qafzeh	XVII; XIX	92 ± 5	17	^8,37,38^

## Results

**Figure 2:**
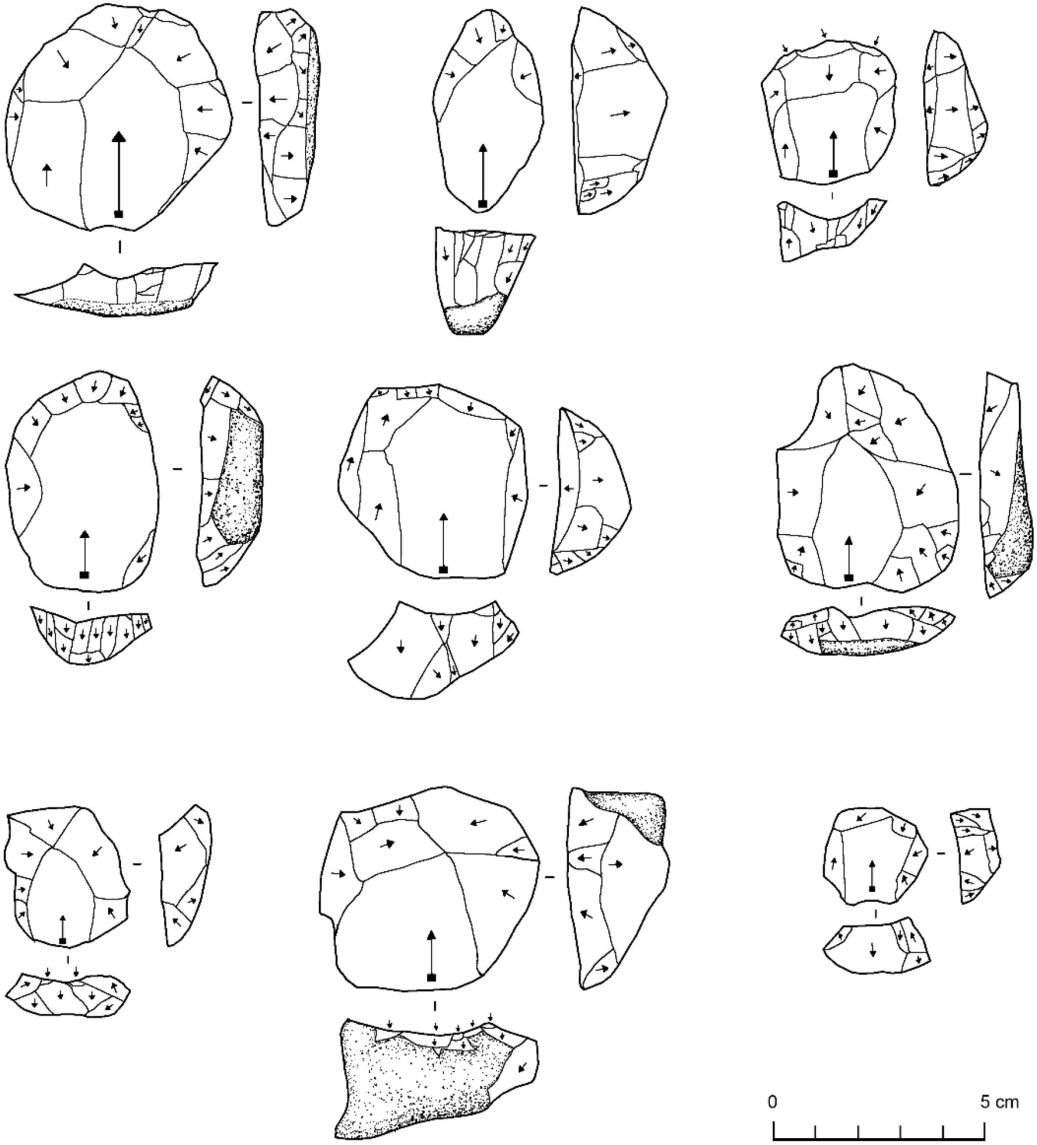
Examples of centripetal Levallois cores from (top) Aduma (A5), (middle) Mundafan (MDF61), and bottom (Al Wusta).

We studied a total of 297 Levallois cores dating from MIS 5 from six sites, including those in eastern African (Aduma A5 [A5]; Omo Kibish Birds Nest Site [BNS]), Arabia (Al Wusta and Mundafan [MDF61]) and the Levant (Qafzeh; Skhul) (Table 1; Fig. 2). Some basic differences exist between the sites and the assemblages, which may structure the results of the ensuing analyses. Firstly, the two Levantine sites have yielded stone tool assemblages from the occupation of cave sites, rather than open air settings. Secondly, the overall size of stone tool assemblages and the number of Levallois cores identified in each assemblage differs. Thirdly, selective curation practices at Skhul may result in the larger artefact sizes retained within this assemblage. Our examination of both the univariate and multivariate variability of the dataset helps to constrain the influence of these factors.

### Univariate analyses

#### Raw material use

There is little evidence to suggest significant transport of raw materials, most of which appear sourced within the immediate environs (ca. 10 km) from the sites, although the use of non-local resources is noted at Skhul^22^ and may be present elsewhere. Cores studied here are predominately made on chert (total = 70%); it is the exclusive raw material used at MDF61, Qafzeh and Skhul, and it comprises 78% of cores from Al Wusta and 46% of cores from BNS (Table SI1.1.1). Chert appears at a much lower frequency in Aduma (6%), which is instead predominately comprised of basalt cores alongside other igneous materials (78%). Lower proportions of both quartz and quartzites comprise the remaining cores from Al Wusta. As a gross indicator of differential use of these materials, we examined the relationship between core weight and raw material, which indicates significant variability between raw material types (see SI1.1). Crypto-crystaline silca (n = 10), obsidian (n = 11) and quartz (n = 3) cores all exhibit low weights in contrast to the more numerous igneous (n = 65) and chert (n = 210) cores, as well as rarer quartzite (n = 9) cores (Table SI1.1.2). We recognise that raw material availability and choices can have substantive impacts of reduction trajectories but argue that the imposition of form through the use of Levallois methods may have more prominent impacts on core morphologies, noting that in each case, crypto-crystaline silica, obsidian and quartz cores occur in assemblages with other material types.

#### Reduction intensity

A number of variables offer general indices of reduction intensity, such as core weight and maximum dimensions. As cores are inherently discarded at the end of a reduction sequence, they may preserve useful information regarding discard thresholds for complete Levallois cores, though may have substantially transformed the size of the initial clast. Preliminary examination suggests considerable overlap in alternate assessments of reduction intensity between core weight and other variables; for the ease of replication, core weight is examined here. Significant groupwise differences in core weight are identified between sites and regions. Pairwise tests indicate cores from BNS and Al Wusta are smaller than cores from other sites, but are comparable to one another, whereas cores from Skhul are larger than other sites except for Qafzeh (Table SI1.2.1). This leads to a common range of variability observed in eastern Africa and Arabia that differs significantly from the Levant (Table SI1.2.2), where Levallois cores appear to have been discarded in larger sizes (Table SI1.2.3).

#### Core shaping

Significant variability is observed between sites and regions in terms core size variables, largely replicating the pattern identified above for reduction intensity. Further variability is observed amongst core shaping indices which are reviewed in greater detail here (see SI1.3). Core flattening differs at both inter-site and inter-regional scales. The ratio of core width to thickness is significantly lower at Al Wusta than all other sites, with values from A5 significantly lower than the remaining sites, whereas flatter cores are seen at MDF61 in contrast to BNS (Table SI1.3.1). As a result, Levantine and Arabian cores are typically flatter than those from eastern Africa (Tables SI1.3.2–3). Core elongation, the ratio of length to width, varies significantly between regions (Table SI1.3.4) but not sites, with cores from the Levant (Skhul; Qafzeh) presenting lower elongation values than eastern Africa or Arabia (Table SI1.3.5). Significant pairwise differences indicate cores from MDF61 and BNS presenting more flaring proximal margins than A5, Al Wusta and Qafzeh, with Skhul also showing more flared proximal margins than A5 and Qafzeh (Table SI1.3.6). At a regional level, eastern African cores have more parallel proximal margins than Arabian cores (Tables SI1.3.7–8). The distal shape of cores from Qafzeh is less convergent that cores from A5, BNS, MDF61 and Skhul, whereas cores from A5 are more convergent that those from Al Wusta and MDF61, with cores from Skhul also more convergent than those from Al Wusta (Table SI1.3.9). Differences in distal core shape are significant between eastern Africa and Arabia (Tables SI1.3.10–11). Significant differences in the count of scars on the flaking face are observed between sites (Tables SI1.3.12–13) and regions (Tables SI1.3.14–15). Levantine sites show the largest count of scars, with Al Wusta and A5 showing the lowest average scar counts of 6.3 and 7.1 respectively. Flaking face shaping is heavily dominated by centripetal (87%) or sub-centripetal (9%) scar patterns, with rare examples of bidirectional (3%), unidirectional (n = 2) or converging (n = 1) scar patterns. Significant differences in the proportions of centripetal/sub-centripetal to alternate scar patterns are observed between sites and regions, with Levantine sites showing a notable presence of alternate dorsal scar patterns (14%), which are rarer in Arabia (6%) and absent in eastern Africa (Table SI1.3.12).

#### Striking platform preparation

Differences occur at both a site and regional level in platform types (see SI1.4). In particular, more dihedral platforms appear at Qafzeh than all other sites combined, whereas the two Levantine lack partially cortical platforms that are present in low frequencies in the eastern African and Arabian sites; in all cases, however, multi-facetted platforms are the dominant platform type (Table SI1.4.1). Significant differences in platform width are evident between sites (Table SI1.4.2) and regions (Table SI1.4.3). Al Wusta and BNS share platform widths that are smaller than all other sites, with A5 also showing smaller platforms than MDF61, whereas platform widths at Skhul are larger than all assemblages (Table SI1.4.4). Eastern African platform widths are smaller than Arabia, with both smaller than the Levant, replicating the pattern of reduction intensity identified above.

#### Flake production

Differences in final scar sizes replicate the common pattern of larger cores in the Levant with smaller cores in eastern Africa; here focus is placed on alternate flake production attributes (see SI1.5). Qafzeh shows significantly lower angles between the platform and final scar than Al Wusta, A5 and MDF61 (Table SI1.5.1), and as a result, Levantine platform angles are significantly lower than those from Africa and Arabia (Tables SI1.5.2–3). Flake scars from Qafzeh are less elongate than all sites other than MDF61, whereas notably elongate scars from A5 are significantly different from those at MDF61 and Skhul (Table SI1.5.4), and as a result, eastern African scars are more elongate than those from Arabia or the Levant (Tables SI1.5.5–6). The proximal margins of Levallois flake scars from A5 are significantly more convergent than those from all other sites, with both Skhul and Qafzeh typically showing flaring convergent margins more frequently than either Al Wusta or MDF61 (Table SI.1.5.7). Overall, average Levantine scars show flaring proximal margins, with more parallel scars observed in Arabia whereas African scars tend to be more convergent (Tables SI1.5.8–9). The distal margins from A5 are also notably convergent in contrast to Qafzeh and MDF61, with Skhul also showing more convergent distal margins than Qafzeh (Table SI1.5.10). At a regional level, Arabian scar distal margins are more parallel than those from eastern Africa (Tables SI1.5.11–12). Skhul shows the highest proportions of flake removals with feather terminations, which varies significantly between sites, and contrasts with Qafzeh, where just more than half of the flake scars show feather terminations (Table SI1.5.13), although no significant differences are observed at a regional scale. Significant differences in scar area to flaking area are identified between sites and regions, with scars on cores from Skhul covering a greater proportion of the flaking surface (Table SI1.5.14), leading to a significant difference between the Levant on one hand, and Arabia and eastern Africa on the other (Tables SI1.5.15–16).

### Multivariate analysis

We used principal components analyses (PCA) to examine the role of alternate metric attributes in structuring variability across the core dataset (see Methods; SI2.1). Preliminary results complement findings from univariate analyses, indicating that variability within the dataset predominately derives from size attributes, spanning alternate reduction domains (Table SI2.1.1). The first principal component accounts for ~ 41% of metric attribute variability and is driven by size variables, with broadly comparable contributions from core weight, maximum dimensions, core axial length and widths, platform width and, to a lesser extent, scar length and widths. Gross differences in Levallois core size may reflect interconnecting factors of raw material availability, reduction intensity, discard thresholds, and patterns of skill, although the role of sampling practices cannot be excluded. The second principal component accounts for ~ 12% of metric attribute variability and is driven by shape variables, particularly core and scar distal shape and scar elongation. The shape variability identified by the principal components analysis is independent of this size variability and may better reflect idiosyncratic choice within a Levallois knapping sequence. The first two principal components, which account for over half of the metric attribute dataset variability, show significant differences between assemblages and between regions (Tables SI2.1.2–7). Below, we examine patterns of Levallois core morphological variability within specific domains of technological behaviour, examining imposed shape and flake production.

#### Core shape

**Figure 3:**
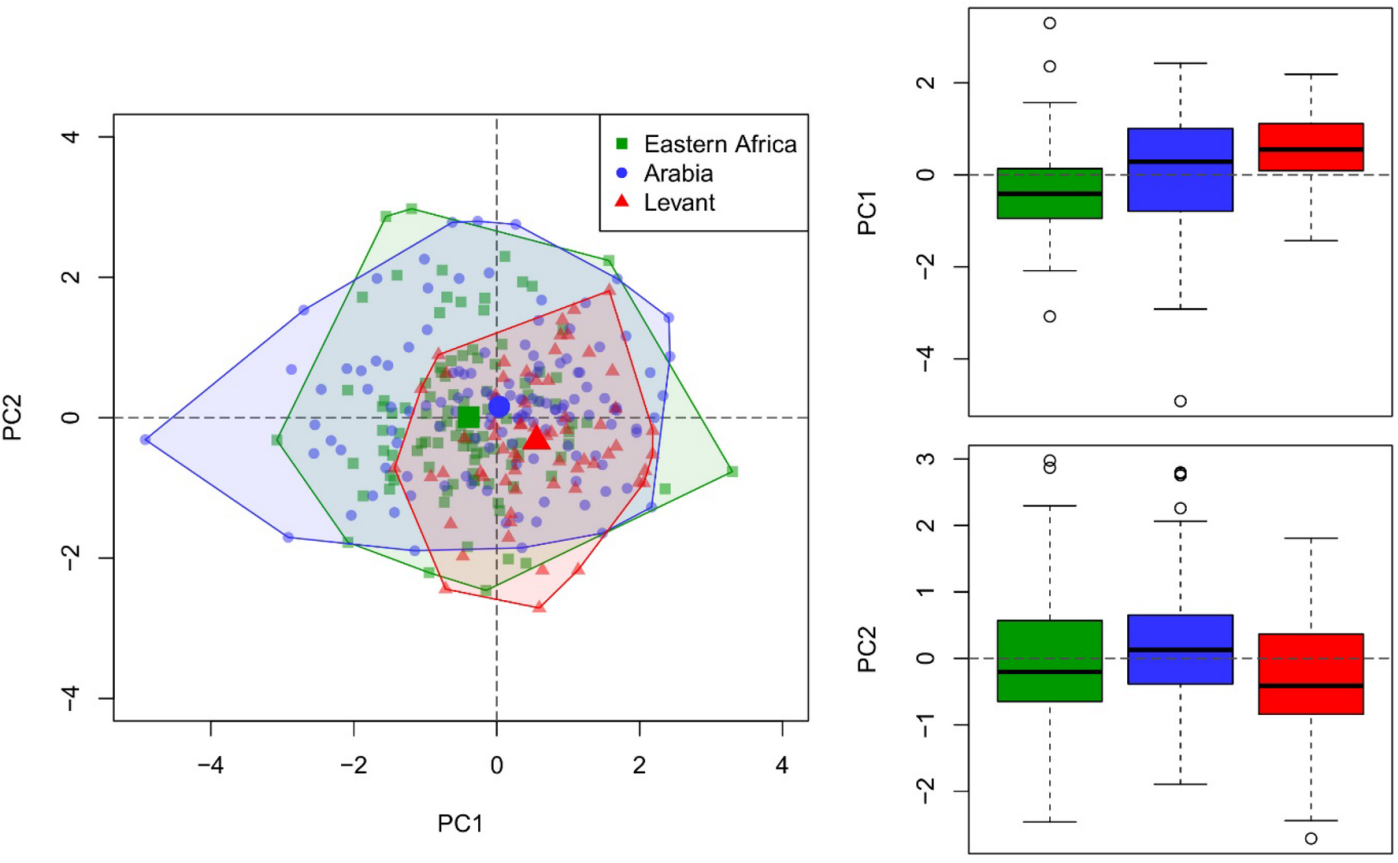
Biplot (left) and boxplots (right) of first two principal components identified in analysis of core shape which account for 61% of identified variability and differ significantly between regions.

Following preliminary evaluation and previous research^32,39^, we examined core shaping, including weight, as a general indicator of core size variables, alongside core elongation, core flattening and core distal shape, using PCA (SI2.2). The first two principal components account for ~ 61% of the variability in the dataset (Table SI2.2.1). The first component (~ 35% variability) is primarily driven by core elongation, which is positively correlated to distal shape but negatively correlated to core flattening and size (Fig. 3). This indicates that larger, flatter cores are less elongate with straighter or flaring distal margins. The second component (~ 26% variability) is primarily driven by differences in distal core shape, which is positively correlated with all other variables, most notably maximum dimension, indicating that smaller cores have more convergent distal margins. Both principal components vary significantly between assemblages and regions (Tables SI.2.2.2–9). Stepwise changes in average values can be identified for the first principal component from eastern Africa (mean = -0.38) to Arabia (mean = 0.03) and then the Levant (mean = 0.56), whereas for the second principal component, the Levant (mean = -0.33) differs from Arabia (mean = 0.16) but not eastern Africa (mean = 0.01), which are largely comparable. Notable variability can be identified within regions, and particularly Arabia, where values for the first principal component for Al Wusta are more comparable to eastern African assemblages than MDF61, which is more comparable to the Levantine assemblages.

#### Flake production

**Figure 4:**
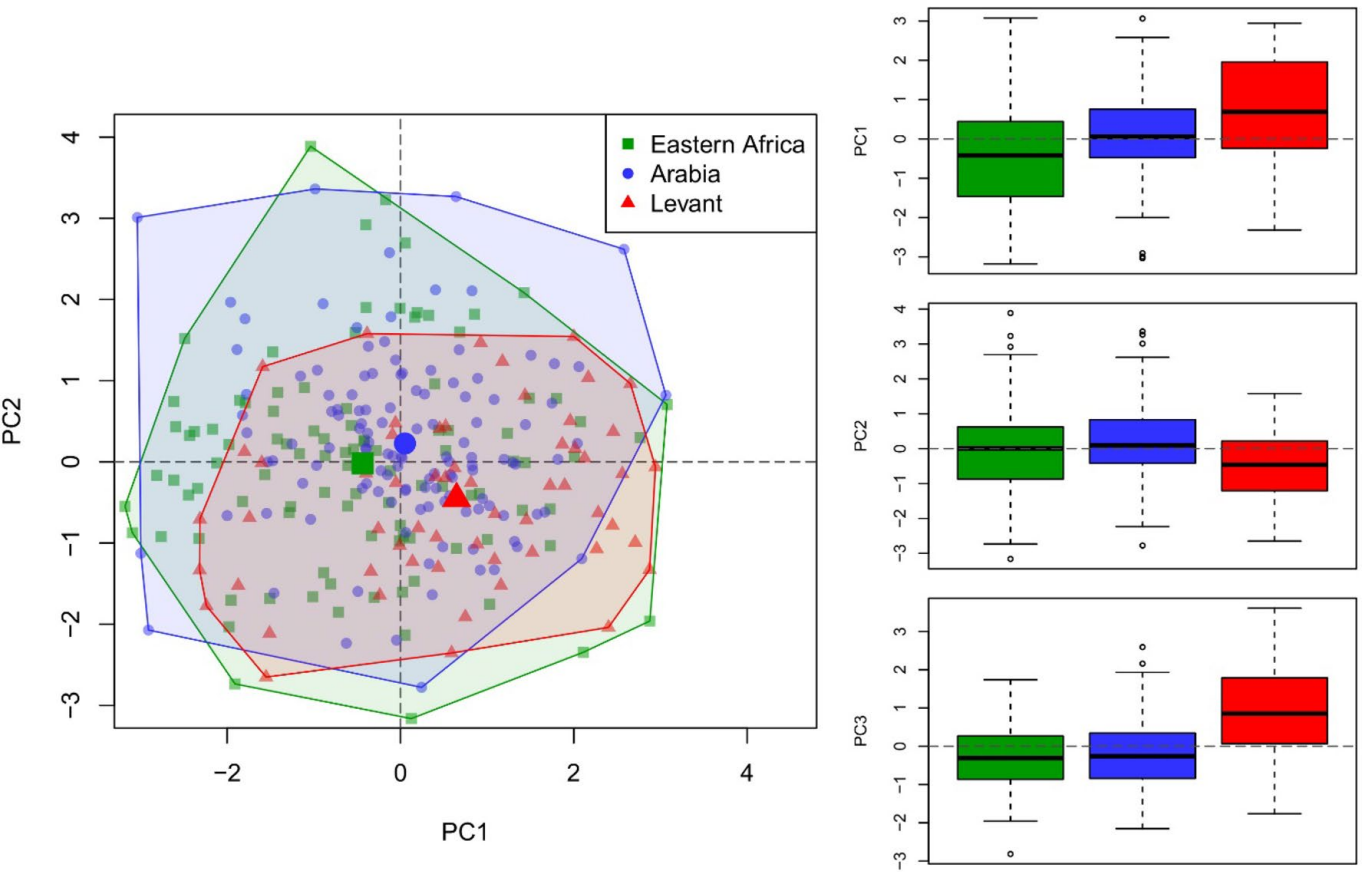
Biplot of the first two principal components (left), and boxplots of the first three principal components (right) identified in analysis of flake production which account for 53% of identified variability and differ significantly between regions.

We examined flake production, including weight, core distal shape, platform angle, scar count on the flake production surface, Levallois scar elongation, proximal and distal shape, and the ratio of scar to flake production surface area, using PCA (SI2.3). The first three principal components account for ~ 53% of the variability in the dataset (Table SI2.3.1). The first component (~ 23% variability) is primarily driven by Levallois scar distal shape, which correlates positively with scar elongation, but shows negative correlation to flaking surface scar counts (Fig. 4). This indicates that more convergent scars are typically more elongate with fewer scars present on the flaking surface. The second component (~ 17% variability) is primarily driven by positive correlations between core weight, core distal shape and flaking surface scar count, indicating larger cores had more convergent distal margins and were shaped by a larger count of scars. The third component (~ 14% variability) is driven by the ratio of scar to flaking surface area, which is negatively correlated to platform angle and, to a lesser extent, to proximal scar shape. This indicates flakes that removed larger proportions of the flaking surface had lower platform angles and parallel or flaring proximal margins. Significant variability is observed across these three principal components between assemblages and regions (Tables SI2.3.2–13). Eastern African cores exhibit lower scores for PC1 (-0.44) than Arabia (0.05), which are in turn lower than those from the Levant (0.66). For PC2, Levant cores (mean = -0.42) return significantly lower values than those from Arabia (mean = 0.22), whereas for PC3, Levantine cores (mean = 0.89) return significantly higher values than either Arabia (mean = -0.21) or eastern Africa (mean = -0.25).

#### Explaining regional differences in Levallois core morphology

Substantial overlaps in Levallois core morphologies can be identified between assemblages from eastern Africa, Arabia, and the Levant in MIS 5, but the analysis above indicates the presence of significant differences between these regions. In order to explore the potential sources of this variability, we examined correlations between the differences in core shaping and flake production identified above and alternate factors relating to each site location, following^40^ (SI2.4). We use cost path distances to examine the extent to which variability amongst Levallois cores may be explained by isolation by distance, which may support a pattern of more direct cultural transmission between closer sites. As distant sites may be situated in drastically different landscapes and resource bases, we pair this with examination of differences in geographic and environmental site contexts to constrain the extent to which such differences can explain technological. Here, we do not presume a direct relationship between Levallois core reduction and the individual parameters examined, but rather acknowledge how they may broadly impact stone tool reduction and use choices.

Distance matrices were calculated between sites for differences in altitude, terrain roughness, and modelled temperature and precipitation for MIS 5 over 5 km and 50 km radii around each site, alongside cost-path distances between sites. We employed multiple-matrix regressions to identify the extent to which each of these variables show significant and independent correlation to the variability we identify in MIS 5 Levallois cores (Table 2; Tables SI2.4.1–4). Amongst the five variables examined, only cost-path distance shows a significant correlation to differences between Levallois cores studied here at a 50 km scale, with a negative correlation to temperature also evident at a 5 km scale. This indicates that patterns of core shaping do not reflect independent influence from wider site landscape, but some impact from the immediate site context may be evident. As a result, incremental changes in core shaping practices between sites and regions may have resulted from cultural practices that do not reflect adaptations to functional constraints but the gradual accumulation of differences in learned behaviours, or cultural drift.

**Table 2:** Results of multiple-matrix regressions between patterns of Levallois core shaping and flake production with alternate examination of geographic parameters at 5 km and 50 km scales, reporting correlation coefficients in bold where significant independent relationships are identified (see SI2.4).

	Core Shaping		Flake Production	
	5 km	50 km	5 km	50 km
Cost Path Distance	**0.024**	**0.024**	**0.031**	**0.034**
Altitude	0.012	0.002	**0.12**	**0.131**
Terrain Roughness	0.008	0.034	− 0.006	**− 0.143**
MIS 5 Temperature	0.029	0.042	**− 0.09**	**− 0.14**
MIS 5 Precipitation	− **0.053**	− 0.074	**0.032**	**0.145**

All five variables examined show significant and independent correlations to differences in Levallois core flake production at a 50 km scale. Larger differences in flake production correlate to larger differences site altitude and temperature but inverse relationships are observed with terrain roughness and precipitation. At the 50 km these variables share comparable strength of relationships to patterns of flake production, but at the 5 km scale, these relationships notably weaken and differences in terrain roughness no longer indicate an independent explanation of variability. A much weaker, though strongly significant, relationship is observed for cost-path distance in both analyses. External factors play much more prominent roles in explaining flake production variability in the core sample, but the independent role of cost-path distances indicates the potential for cultural drift to play a role in shaping differences between regions.

## Discussion

This study provides a quantified appraisal of Levallois core variability between eastern Africa, Arabia, and the Levant in MIS 5, demonstrating the near ubiquitous focus on centripetal reduction schemes with considerable overlaps in core morphology. Analyses of core attribute datasets enable nuanced evaluation of patterns of variability in Levallois reduction schemes that complement those that highlight the potential spread of centripetal Levallois methods by *Homo sapiens* in MIS 5 based on assemblage compositions^10,19,20^, extending the geographic range of other studies of cores in the region^32,41^. While there is extensive shared variability amongst Levallois cores identified here, differences between eastern Africa, Arabia, and the Levant can be resolved in both core shaping and flake production. Differences in core sizes exist within the assemblages studied, but multivariate analyses indicate patterns of variability relating to shape exist independently of size, with directional changes observed from eastern Africa to Arabia and the Levant, from thicker, elongate cores producing elongate, convergent final flakes in eastern Africa to flatter, broader cores producing more squared final flakes in the Levant. Although considerable overlaps in core morphology are observed between sites and regions, a pattern of isolation by distance exists for changing variability in both core shaping and flake production that is discrete from potential geographic and environmental differences between sites. This indicates that differences in Levallois cores spanning eastern Africa, Arabia, and the Levant may illustrate patterns of cultural drift that are independent from functional and technological constraints.

Qafzeh and Skhul clearly differ from the eastern African and Arabian sites as both are cave sites that are found in distinct topographic and ecological landscapes, which may indicate significant roles for patterns of mobility and environmental engagement to explain the patterns that we identify. Patterns of mobility are widely acknowledged to play a critical role in structuring stone tool use and discard, as well as wider patterns of behaviour amongst Palaeolithic populations^42–44^. For instance, Wallace and Shea^44^ have argued that the core assemblage from Qafzeh is consistent with occupation as part of a residential mobility pattern, and it may be the case that the eastern African and Arabian assemblages record wider ranges of variability in site uses within a similar mobility system. Beyond the immediate differences in site occupations, multiple matrix regressions help to identify that geographic and environmental factors may directly and independently contribute to patterns of variability that are most acute for flake production practices and reflect broad features of the landscape. Given the demonstrated focus on centripetal reduction systems and discrete patterns of isolation by distance independent of other geographic or environmental features, we highlight the potential for sequential difference in Levallois core shapes from eastern African to Arabia to the Levant, reflecting gradual behavioural changes through processes of cultural transmission.

The six assemblages studied here share a common focus on centripetal Levallois reduction schemes. The study of varying frequencies of alternate Levallois reduction schemes have historically been a major focus of study in the Levantine Middle Palaeolithic^45–47^, exerting some influence on debates regarding the nascent Arabian record^19^, but largely standing in contrast to examination of the eastern Africa Middle Stone Age^48–50^. In the Levant, the predominant focus on centripetal Levallois reduction typically differentiates the mid-Middle Palaeolithic (also described as Interglacial Middle Palaeolithic or Tabun-C like assemblages), from earlier (late Middle Pleistocene Middle Palaeolithic or Tabun-D like) and later (MIS 4/3 or Tabun-B like) Middle Palaeolithic assemblages^45–47^, although centripetal reduction schemes are known from the terminal Middle Palaeolithic, such as at Kebara^51^. Mid-Middle Palaeolithic assemblages in the region remain scarce, and have largely not been subject to detailed or comparative study, with the recent excavations and analyses of Nesher Ramla a welcome exception^20,52^. Some researchers in the region have suggested some technological continuity between early and mid-Middle Palaeolithic technologies^53^, with changes in the archaeological record potentially reflecting shifts in patterns of land-used and population densities^54^. A recent study has challenged this, arguing the absence of centripetal Levallois technology in the Levant prior to the mid-Middle Palaeolithic does not support a continuous, regional development but rather indicates an origin outside the region^20^. The considerable similarity we document between centripetal Levallois cores across the Levant, Arabia and eastern Africa supports this analysis. Moreover, this study directly complements the focus on MIS 5 for the appearance of centripetal Levallois dominated assemblages across these regions highlighted by Prévost and Zaidner^20^.The earlier yet sporadic appearance of such assemblages in east and north-east Africa is potentially indicative of a source region^20^ (Fig. 1), consistent with the sequential regional variability in core form we illuminate.

This study demonstrates directional changes in technological practice from eastern Africa to Arabia and the Levant during MIS 5 that exist alongside a common focus on centripetal Levallois reduction and overlapping core morphologies. Vertical cultural transmission from a shared source is likely to be accentuated within small and mobile hunter-gatherer populations^18,29^. The directional patterns we identify may be indicative of cultural drift, relating to idiosyncratic features of knapping practice that were not technologically constrained or sensitive to environmental parameters, and thus may help resolve a shared cultural inheritance. Considerable focus has been placed upon identifying discrete technological features, particularly the appearance of Nubian Levallois technology, to illuminate patterns of human dispersals from lithic assemblages^55,56^. However, Nubian Levallois cores typically occur in low proportions in most assemblages where present and have a questionable association with *Homo sapiens* fossils^57^, as well as having recently been identified in association with Neanderthal populations^30^. We contend that further focus on centripetal Levallois technologies is best placed to illuminate patterns of cultural inheritance at the interface of Africa and Eurasia in MIS 5, capitalising on its dominance within assemblages directly associated with *Homo sapiens* and leveraging this strength to document communities of technical practice and enable parallel examination of behavioural evolution for a key phase of demographic change.

## Methods

Following previous research examining Levallois reduction sequences through attribute analyses^31^, including studies of inter-regional core variability in Middle Palaeolithic and Middle Stone Age (^32,39^), we recorded whether cores were complete, percentages of cortical coverage, weight, maximum dimensions, axial dimensions, platform dimensions, platform angle and dimensions of the last Levallois removal, platform type, termination type, flaking face scar pattern, the count of scars greater than 5 mm, and whether the core meets all descriptive features of Levallois technology, following Boeda^28^. Additional variables were generated as indices from these measurements, including elongation (ratio of L:W), distal shapes (ratio of Medial : Distal Width) and flattening (ratio of Medial Width : Medial Thickness). Full descriptions of these attributes, how they are recorded, and how they reflect choices made through stone reduction sequences are presented by ^58^. Only cores that were complete, clearly preserved the final sequence of Levallois flaking, and for which all variables could be recorded were retained for analysis. Basic descriptions of the core assemblages are provided using measures of central tendency and variability at a univariate level for all metric variables, as well as comparisons of frequency for categorical datasets. Statistical analysis was conducted using R 4.0.1^59^. Differences between sites are examined at groupwise levels using Chi-squared and Kruskal–Wallis tests and at pairwise levels using Fisher’s and Wilcox tests, employing Benjamini–Hochberg p-value adjustments where necessary. Results reported as significant in the main text fall below a standard alpha = 0.05 threshold, with full results of statistical tests reported in SI. Variables were selected for multivariate analyses to offer close comparisons to other, related studies^29^. Datasets were normalised using the BestNormalize package^60^, with centred and scaled datasets subject to Principal Components Analysis using *prcomp*. Multiple matrix regressions were undertaken following methods set out by ^40^, including calculation of cost path distances using the *gdistance* package^61^, and calculation of histogram-based dissimilarity matrices from freely available spatial datasets, including the SRTM DEM^25^ and modelled precipitation and temperature for the last Interglacial^62^, using the *HistDAWass* package ^63^. Multiple-matrix regressions were calculated using 9999 permutations using the *phytools* package ^64^.

## Supplementary Information

Supplementary Information 1.

Supplementary Information 2.

Supplementary Information 3.

## Data Availability

The analytical code and full dataset used in the analyses are presented as SI3 and SI4 respectively.
