# Supplementary material for: Directional changes in Levallois core technologies between Eastern Africa, Arabia, and the Levant during MIS 5
Source: Sci Rep. 2021 Jun 1;11:11465. doi: 10.1038/s41598-021-90744-z (PMC8169925; doi:10.1038/s41598-021-90744-z)
Supplement: Supplementary file 1 — Supplementary Information 1. [file 41598_2021_90744_MOESM1_ESM.docx]

**Directional changes in Levallois core technologies between Eastern Africa, Arabia and the Levant during MIS 5**

James Blinkhorn^1,2^, Huw S. Groucutt^3,4,5^, Eleanor M. L. Scerri^1,5,6^, Michael D. Petraglia^4,7,8,9^, Simon Blockley^2^

^1^Pan African Evolution Research Group, Max Planck Institute for the Science of Human History

^2^Centre for Quaternary Research, Department of Geography, Royal Holloway, University of London

^3^Extreme Events Research Group, Max Planck Institutes for Chemical Ecology, The Science of Human History, and Biogeochemistry, Hans‑Knöll‑Strasse 8, 07745 Jena, Germany.

^4^Department of Archaeology, Max Planck Institute for the Science of Human History, Kahlaische Strasse 10, 07745 Jena, Germany.

^5^Institute of Prehistoric Archaeology, University of Cologne, Cologne, 50931, Germany

^6^Department of Classics and Archaeology, University of Malta, Msida 2080, MSD, Malta

^7^Human Origins Program, Smithsonian Institution, Washington, DC 20560, USA.

^8^School of Social Science, The University of Queensland, Brisbane, QLD 4072, Australia.

^9^Australian Research Centre for Human Evolution, Environmental Futures Research Institute, Griffith University, Brisbane, Australia

Corresponding author: J Blinkhorn; email: blinkhorn@shh.mpg.de

**Supplementary Information 1: Results of Univariate Analyses**

**SI1.1 Raw Material Use**

Table SI1.1.1: Raw Material use in core assemblages studied.

|  | CCS | Chert | Igneous | Obsidian | Quartz | Quartzite |
| --- | --- | --- | --- | --- | --- | --- |
| A5 | 1 | 5 | 62 | 11 | 0 | 0 |
| Al Wusta | 0 | 43 | 0 | 0 | 3 | 9 |
| BNS | 9 | 11 | 4 | 0 | 0 | 0 |
| MDF61 | 0 | 88 | 0 | 0 | 0 | 0 |
| Qafzeh | 0 | 17 | 0 | 0 | 0 | 0 |
| Skhul | 0 | 47 | 0 | 0 | 0 | 0 |

Results of groupwise testing of raw material use by weight: Kruskal-Wallis chi-squared = 52.029, df = 5, p-value = 5.322e-10

Table SI1.1.2: Descriptive statistics of core weight by raw material.

|  | n | mean | sd | median | trimmed | mad | min | max | range | skew | kurtosis | se |
| --- | --- | --- | --- | --- | --- | --- | --- | --- | --- | --- | --- | --- |
| CCS | 10 | 11.34 | 4.41 | 12.35 | 11.80 | 4.74 | 3.10 | 15.90 | 12.80 | -0.56 | -1.24 | 1.39 |
| Chert | 210 | 47.25 | 51.08 | 31.15 | 37.71 | 22.02 | 3.20 | 397.20 | 394.00 | 3.76 | 19.52 | 3.52 |
| Igneous | 65 | 63.16 | 63.38 | 42.20 | 50.96 | 29.95 | 2.60 | 341.90 | 339.30 | 2.31 | 6.07 | 7.86 |
| Obsidian | 11 | 5.26 | 3.76 | 4.10 | 4.79 | 2.82 | 1.40 | 13.40 | 12.00 | 0.93 | -0.46 | 1.13 |
| Quartz | 3 | 21.60 | 12.06 | 17.40 | 21.60 | 7.71 | 12.20 | 35.20 | 23.00 | 0.31 | -2.33 | 6.96 |
| Quartzite | 9 | 75.00 | 75.63 | 31.40 | 75.00 | 42.25 | 2.90 | 212.40 | 209.50 | 0.57 | -1.39 | 25.21 |

**SI1.2 Reduction Intensity**

Table SI1.2.1: Results of multiple pairwise Wilcox tests for differences in core weight between sites.

|  | A5 | Al Wusta | BNS | MDF61 | Qafzeh |
| --- | --- | --- | --- | --- | --- |
| Al Wusta | 0.02988 | - | - | - | - |
| BNS | 0.00671 | 0.37303 | - | - | - |
| MDF61 | 0.28477 | 8.90E-05 | 6.20E-06 | - | - |
| Qafzeh | 0.15031 | 0.00438 | 0.00078 | 0.31366 | - |
| Skhul | 0.02104 | 7.70E-06 | 2.30E-06 | 0.02988 | 0.93942 |

Table SI1.2.2: Results of multiple pairwise Wilcox tests for differences in core weight between regions.

|  | Eastern Africa | Arabia |
| --- | --- | --- |
| Arabia | 0.24 | - |
| Levant | 9.90E-05 | 9.90E-05 |

Table SI1.2.3: Descriptive statistics of core weight by site.

|  | n | mean | sd | median | trimmed | mad | min | max | range | skew | kurtosis | se |
| --- | --- | --- | --- | --- | --- | --- | --- | --- | --- | --- | --- | --- |
| A5 | 78 | 52.44 | 61.668 | 29.15 | 40.514 | 29.207 | 1.4 | 341.9 | 340.5 | 2.468 | 7.102 | 6.982 |
| Al Wusta | 55 | 38.34 | 61.989 | 17.4 | 24.711 | 15.270 | 2.9 | 388.3 | 385.4 | 3.825 | 16.95 | 8.358 |
| BNS | 24 | 23.07 | 27.004 | 15.5 | 16.52 | 10.229 | 3.1 | 117.3 | 114.2 | 2.547 | 5.534 | 5.5120 |
| MDF61 | 87 | 46.01 | 33.559 | 35.3 | 40.316 | 17.494 | 7.7 | 191 | 183.3 | 1.865 | 3.788 | 3.5979 |
| Qafzeh | 17 | 78.06 | 74.920 | 46.4 | 72.88 | 42.105 | 12.5 | 221.4 | 208.9 | 0.995 | -0.732 | 18.170 |
| Skhul | 47 | 60.77 | 58.471 | 46.7 | 50.743 | 27.131 | 15.5 | 397.2 | 381.7 | 4.133 | 20.584 | 8.528 |

**SI1.3: Core Shaping**

Table SI1.3.1: Results of multiple pairwise Wilcox tests for differences in core flattening between sites.

|  | A5 | Al Wusta | BNS | MDF61 | Qafzeh |
| --- | --- | --- | --- | --- | --- |
| Al Wusta | 0.02573 | - | - | - | - |
| BNS | 0.00477 | 0.00028 | - | - | - |
| MDF61 | 3.70E-12 | 3.70E-12 | 0.00477 | - | - |
| Qafzeh | 0.00011 | 1.80E-05 | 0.07993 | 0.58785 | - |
| Skhul | 2.90E-06 | 1.70E-07 | 0.19346 | 0.06365 | 0.48994 |

Table SI1.3.2: Results of multiple pairwise Wilcox tests for differences in core flattening between regions.

|  | Eastern Africa | Arabia |
| --- | --- | --- |
| Arabia | 0.00099 | - |
| Levant | 4.90E-07 | 0.11875 |

Table SI1.3.3: Descriptive statistics of core flattening by site.

|  | n | mean | sd | median | trimmed | mad | min | max | range | skew | kurtosis | se |
| --- | --- | --- | --- | --- | --- | --- | --- | --- | --- | --- | --- | --- |
| A5 | 79 | 1.981772 | 0.581858 | 1.87 | 1.929846 | 0.504084 | 1.07 | 3.78 | 2.71 | 0.822396 | 0.326981 | 0.065464 |
| Al Wusta | 50 | 1.7492 | 0.683881 | 1.53 | 1.66075 | 0.674583 | 0.89 | 4.15 | 3.26 | 1.207035 | 1.620474 | 0.096715 |
| BNS | 24 | 2.408333 | 0.683296 | 2.325 | 2.34 | 0.555975 | 1.41 | 4.53 | 3.12 | 1.219964 | 1.644025 | 0.139477 |
| MDF61 | 86 | 3.008023 | 0.981577 | 2.775 | 2.929571 | 0.874734 | 1.42 | 5.7 | 4.28 | 0.716104 | -0.0446 | 0.105846 |
| Qafzeh | 17 | 2.813529 | 0.760386 | 2.73 | 2.792 | 0.696822 | 1.66 | 4.29 | 2.63 | 0.471386 | -0.83696 | 0.184421 |
| Skhul | 46 | 2.670217 | 0.842297 | 2.535 | 2.597105 | 0.659757 | 1.18 | 5.17 | 3.99 | 1.021055 | 1.269998 | 0.12419 |

Table SI1.3.4: Results of multiple pairwise Wilcox tests for differences in core elongation between regions.

|  | Eastern Africa | Arabia |
| --- | --- | --- |
| Arabia | 0.438 | - |
| Levant | 0.012 | 0.076 |

Table SI1.3.5: Descriptive statistics of core elongation by region.

|  | n | mean | sd | median | trimmed | mad | min | max | range | skew | kurtosis | se |
| --- | --- | --- | --- | --- | --- | --- | --- | --- | --- | --- | --- | --- |
| Eastern Africa | 102 | 1.042745 | 0.188129 | 1.03 | 1.035244 | 0.163086 | 0.48 | 1.62 | 1.14 | 0.35924 | 1.173064 | 0.018628 |
| Arabia | 129 | 1.037054 | 0.234888 | 1.02 | 1.019905 | 0.192738 | 0.57 | 2.24 | 1.67 | 1.26897 | 4.163272 | 0.020681 |
| Levant | 64 | 0.964531 | 0.146709 | 0.98 | 0.962885 | 0.133434 | 0.47 | 1.36 | 0.89 | -0.19462 | 1.067698 | 0.018339 |

Table SI1.3.6: Results of multiple pairwise Wilcox tests for differences in core proximal shape between sites.

|  | A5 | Al Wusta | BNS | MDF61 | Qafzeh |
| --- | --- | --- | --- | --- | --- |
| Al Wusta | 0.11819 | - | - | - | - |
| BNS | 0.00285 | 0.02447 | - | - | - |
| MDF61 | 5.20E-06 | 0.00362 | 0.82805 | - | - |
| Qafzeh | 0.72822 | 0.25745 | 0.03093 | 0.00898 | - |
| Skhul | 0.00095 | 0.05611 | 0.25745 | 0.14574 | 0.02058 |

Table SI1.3.7: Results of multiple pairwise Wilcox tests for differences in core proximal shape between regions.

|  | Eastern Africa | Arabia |
| --- | --- | --- |
| Arabia | 0.0046 | - |
| Levant | 0.069 | 0.2164 |

Table SI1.3.8: Descriptive statistics of core proximal shape by site.

|  | n | mean | sd | median | trimmed | mad | min | max | range | skew | kurtosis | se |
| --- | --- | --- | --- | --- | --- | --- | --- | --- | --- | --- | --- | --- |
| A5 | 79 | 0.924 | 0.167 | 0.940 | 0.927 | 0.163 | 0.480 | 1.250 | 0.770 | -0.260 | -0.317 | 0.019 |
| Al Wusta | 50 | 0.879 | 0.154 | 0.885 | 0.873 | 0.156 | 0.570 | 1.280 | 0.710 | 0.338 | -0.188 | 0.022 |
| BNS | 24 | 0.785 | 0.168 | 0.725 | 0.770 | 0.119 | 0.550 | 1.190 | 0.640 | 0.814 | -0.385 | 0.034 |
| MDF61 | 86 | 0.786 | 0.182 | 0.755 | 0.775 | 0.156 | 0.390 | 1.470 | 1.080 | 0.883 | 1.575 | 0.020 |
| Qafzeh | 17 | 0.912 | 0.136 | 0.930 | 0.915 | 0.044 | 0.620 | 1.170 | 0.550 | -0.282 | -0.162 | 0.033 |
| Skhul | 46 | 0.811 | 0.132 | 0.810 | 0.813 | 0.089 | 0.460 | 1.120 | 0.660 | -0.223 | 0.159 | 0.020 |

Table SI1.3.9: Results of multiple pairwise Wilcox tests for differences in core distal shape between sites.

|  | A5 | Al Wusta | BNS | MDF61 | Qafzeh |
| --- | --- | --- | --- | --- | --- |
| Al Wusta | 0.014 | - | - | - | - |
| BNS | 0.6565 | 0.1196 | - | - | - |
| MDF61 | 0.0311 | 0.1986 | 0.337 | - | - |
| Qafzeh | 0.0014 | 0.2721 | 0.0204 | 0.0215 | - |
| Skhul | 0.9877 | 0.0353 | 0.7269 | 0.1986 | 0.0167 |

Table SI1.3.10: Results of multiple pairwise Wilcox tests for differences in core distal shape between regions.

|  | Eastern Africa | Arabia |
| --- | --- | --- |
| Arabia | 0.0034 | - |
| Levant | 0.1709 | 0.5082 |

Table SI1.3.11: Descriptive statistics of core distal shape by site.

|  | n | mean | sd | median | trimmed | mad | min | max | range | skew | kurtosis | se |
| --- | --- | --- | --- | --- | --- | --- | --- | --- | --- | --- | --- | --- |
| A5 | 79 | 1.49 | 0.37 | 1.40 | 1.44 | 0.25 | 0.96 | 3.01 | 2.05 | 1.73 | 3.86 | 0.04 |
| Al Wusta | 50 | 1.39 | 0.59 | 1.23 | 1.28 | 0.24 | 0.89 | 4.72 | 3.83 | 3.78 | 17.66 | 0.08 |
| BNS | 24 | 1.49 | 0.48 | 1.38 | 1.41 | 0.25 | 0.99 | 3.23 | 2.24 | 2.15 | 4.90 | 0.10 |
| MDF61 | 86 | 1.43 | 0.50 | 1.31 | 1.33 | 0.19 | 0.95 | 4.38 | 3.43 | 3.43 | 14.41 | 0.05 |
| Qafzeh | 17 | 1.19 | 0.13 | 1.20 | 1.20 | 0.16 | 0.90 | 1.39 | 0.49 | -0.27 | -0.89 | 0.03 |
| Skhul | 46 | 1.72 | 0.88 | 1.36 | 1.56 | 0.34 | 0.94 | 5.12 | 4.18 | 2.10 | 4.34 | 0.13 |

Table SI1.3.12: Results of multiple pairwise Wilcox tests for differences in scars on flaking surface >5mm between sites.

|  | A5 | AlWusta | BNS | MDF61 | Qafzeh |
| --- | --- | --- | --- | --- | --- |
| AlWusta | 0.1831 | - | - | - | - |
| BNS | 0.00487 | 0.00047 | - | - | - |
| MDF61 | 0.02341 | 0.00055 | 0.11764 | - | - |
| Qafzeh | 6.30E-05 | 1.40E-05 | 0.11978 | 0.00053 | - |
| Skhul | 1.40E-06 | 1.80E-07 | 0.3223 | 0.00024 | 0.42259 |

Table SI1.3.13: Descriptive statistics of scars on flaking surface >5mm by site

|  | n | mean | sd | median | trimmed | mad | min | max | range | skew | kurtosis | se |
| --- | --- | --- | --- | --- | --- | --- | --- | --- | --- | --- | --- | --- |
| A5 | 79 | 7.11 | 2.39 | 7 | 6.83 | 1.48 | 4 | 17 | 13 | 1.37 | 2.60 | 0.27 |
| AlWusta | 44 | 6.32 | 1.83 | 6 | 6.31 | 1.48 | 1 | 10 | 9 | -0.08 | 0.53 | 0.28 |
| BNS | 24 | 10.04 | 5.32 | 8 | 9.30 | 2.22 | 4 | 26 | 22 | 1.49 | 1.45 | 1.09 |
| MDF61 | 86 | 7.85 | 2.28 | 7 | 7.64 | 1.48 | 4 | 14 | 10 | 0.79 | 0.14 | 0.25 |
| Qafzeh | 17 | 11.12 | 3.72 | 11 | 10.93 | 2.97 | 5 | 20 | 15 | 0.49 | -0.16 | 0.90 |
| Skhul | 47 | 10.62 | 4.41 | 10 | 10.10 | 4.45 | 5 | 27 | 22 | 1.42 | 2.60 | 0.64 |

Table SI1.31.4: Results of multiple pairwise Wilcox tests for differences in scars on flaking surface >5mm between regions.

|  | Eastern Africa | Arabia |
| --- | --- | --- |
| Arabia | 0.92 | - |
| Levant | 1.20E-07 | 2.60E-09 |

Table SI1.3.15: Descriptive statistics of scars on flaking surface >5mm by region.

|  | n | mean | sd | median | trimmed | mad | min | max | range | skew | kurtosis | se |
| --- | --- | --- | --- | --- | --- | --- | --- | --- | --- | --- | --- | --- |
| Eastern Africa | 103 | 7.80 | 3.51 | 7 | 7.22 | 1.48 | 4 | 26 | 22 | 2.38 | 7.66 | 0.35 |
| Arabia | 130 | 7.33 | 2.25 | 7 | 7.14 | 1.48 | 1 | 14 | 13 | 0.67 | 0.73 | 0.20 |
| Levant | 64 | 10.75 | 4.22 | 10 | 10.31 | 4.45 | 5 | 27 | 22 | 1.26 | 2.28 | 0.53 |

Table SI1.3.16: Core scar pattern by region.

|  | Centripetal | Other |
| --- | --- | --- |
| Eastern Africa | 97 | 6 |
| Arabia | 130 | 0 |
| Levant | 55 | 9 |

Results of groupwise testing of core scar pattern by region: X-squared = 17.883, df = 2, p-value = 0.0001308

**SI1.4: Striking Platform Preparation**

Table SI1.4.1: Platform type by site

|  | Cortical | Dihedral | Multi-Facet | Plain |
| --- | --- | --- | --- | --- |
| A5 | 3 | 3 | 71 | 2 |
| Al Wusta | 2 | 1 | 38 | 3 |
| BNS | 4 | 0 | 20 | 0 |
| MDF61 | 2 | 1 | 77 | 3 |
| Qafzeh | 0 | 6 | 10 | 1 |
| Skhul | 0 | 0 | 44 | 2 |

Results of groupwise testing of platform type by site: X-squared = 67.666, df = 15, p-value = 1.16e-08

Results of groupwise testing of platform type by region: X-squared = 13.918, df = 6, p-value = 0.03056

Table SI1.4.2: Results of multiple pairwise Wilcox tests for differences in platform width between sites.

|  | A5 | Al Wusta | BNS | MDF61 | Qafzeh |
| --- | --- | --- | --- | --- | --- |
| Al Wusta | 0.00615 | - | - | - | - |
| BNS | 0.00067 | 0.37988 | - | - | - |
| MDF61 | 6.90E-07 | 1.60E-09 | 1.60E-09 | - | - |
| Qafzeh | 0.0893 | 0.00332 | 0.0007 | 0.48002 | - |
| Skhul | 3.80E-13 | 6.60E-12 | 5.20E-10 | 7.40E-09 | 0.0006 |

Table SI1.4.3: Results of multiple pairwise Wilcox tests for differences in platform width between regions.

|  | Eastern Africa | Arabia |
| --- | --- | --- |
| Arabia | 0.00013 | - |
| Levant | 3.20E-14 | 1.30E-09 |

Table SI1.4.4: Descriptive statistics of platform width by site.

|  | n | mean | sd | median | trimmed | mad | min | max | range | skew | kurtosis | se |
| --- | --- | --- | --- | --- | --- | --- | --- | --- | --- | --- | --- | --- |
| A5 | 79 | 24.68 | 8.04 | 24.85 | 24.53 | 6.98 | 6.15 | 57.49 | 51.34 | 0.60 | 2.36 | 0.90 |
| Al Wusta | 44 | 21.40 | 9.40 | 18.54 | 20.38 | 6.49 | 9.19 | 63.62 | 54.43 | 2.11 | 6.96 | 1.42 |
| BNS | 23 | 18.77 | 5.37 | 18.74 | 18.64 | 5.32 | 8.78 | 31.67 | 22.89 | 0.27 | -0.36 | 1.12 |
| MDF61 | 86 | 30.89 | 7.69 | 30.37 | 30.49 | 5.89 | 13.69 | 68.00 | 54.31 | 1.38 | 5.34 | 0.83 |
| Qafzeh | 17 | 29.36 | 10.69 | 28.25 | 29.19 | 10.27 | 11.20 | 50.07 | 38.87 | 0.26 | -0.87 | 2.59 |
| Skhul | 46 | 42.92 | 12.49 | 41.22 | 42.17 | 11.39 | 19.27 | 77.00 | 57.73 | 0.60 | 0.10 | 1.84 |

**SI1.5: Flake Production**

Table SI1.5.1: Results of multiple pairwise Wilcox tests for differences in core platform angle between sites.

|  | A5 | Al Wusta | BNS | MDF61 | Qafzeh |
| --- | --- | --- | --- | --- | --- |
| Al Wusta | 0.335 | - | - | - | - |
| BNS | 0.253 | 0.159 | - | - | - |
| MDF61 | 0.271 | 0.128 | 0.456 | - | - |
| Qafzeh | 0.014 | 0.014 | 0.128 | 0.014 | - |
| Skhul | 0.128 | 0.095 | 0.796 | 0.271 | 0.128 |

Table SI1.5.2: Results of multiple pairwise Wilcox tests for differences in core platform angle between regions.

|  | Eastern Africa | Arabia |
| --- | --- | --- |
| Arabia | 0.8394 | - |
| Levant | 0.0121 | 0.0088 |

Table SI1.5.3: Descriptive statistics of core platform angle by site.

|  | n | mean | sd | median | trimmed | mad | min | max | range | skew | kurtosis | se |
| --- | --- | --- | --- | --- | --- | --- | --- | --- | --- | --- | --- | --- |
| A5 | 79 | 71.03 | 6.85 | 72.00 | 71.37 | 5.93 | 50.00 | 90.00 | 40.00 | -0.51 | 0.78 | 0.77 |
| Al Wusta | 52 | 73.54 | 10.54 | 74.00 | 72.74 | 8.90 | 56.00 | 100.00 | 44.00 | 0.58 | 0.02 | 1.46 |
| BNS | 23 | 69.17 | 6.36 | 70.00 | 69.00 | 7.41 | 60.00 | 82.00 | 22.00 | 0.10 | -0.99 | 1.33 |
| MDF61 | 82 | 70.11 | 5.32 | 70.00 | 70.38 | 5.93 | 53.00 | 81.00 | 28.00 | -0.52 | 0.09 | 0.59 |
| Qafzeh | 17 | 65.12 | 6.07 | 64.00 | 64.93 | 5.93 | 57.00 | 76.00 | 19.00 | 0.42 | -1.20 | 1.47 |
| Skhul | 44 | 68.55 | 8.27 | 68.00 | 68.83 | 7.41 | 50.00 | 85.00 | 35.00 | -0.29 | -0.28 | 1.25 |

Table SI1.5.4: Results of multiple pairwise Wilcox tests for differences in scar elongation between sites.

|  | A5 | Al Wusta | BNS | MDF61 | Qafzeh |
| --- | --- | --- | --- | --- | --- |
| Al Wusta | 0.1131 | - | - | - | - |
| BNS | 0.2848 | 0.9474 | - | - | - |
| MDF61 | 0.0017 | 0.5443 | 0.5443 | - | - |
| Qafzeh | 0.0003 | 0.0282 | 0.1249 | 0.0169 | - |
| Skhul | 0.0145 | 0.5805 | 0.5443 | 0.9474 | 0.0169 |

Table SI1.5.5: Results of multiple pairwise Wilcox tests for differences in scar elongation between regions.

|  | Eastern Africa | Arabia |
| --- | --- | --- |
| Arabia | 0.00245 | - |
| Levant | 0.00025 | 0.1308 |

Table SI1.5.6: Descriptive statistics of scar elongation by site.

|  | n | mean | sd | median | trimmed | mad | min | max | range | skew | kurtosis | se |
| --- | --- | --- | --- | --- | --- | --- | --- | --- | --- | --- | --- | --- |
| A5 | 79 | 1.50 | 0.582 | 1.390 | 1.424 | 0.489 | 0.650 | 4.120 | 3.470 | 1.875 | 5.090 | 0.065 |
| Al Wusta | 44 | 1.29 | 0.486 | 1.170 | 1.265 | 0.460 | 0.340 | 2.410 | 2.070 | 0.462 | -0.381 | 0.073 |
| BNS | 23 | 1.310 | 0.676 | 1.280 | 1.239 | 0.474 | 0.330 | 3.050 | 2.720 | 0.832 | 0.407 | 0.141 |
| MDF61 | 86 | 1.201 | 0.352 | 1.185 | 1.174 | 0.319 | 0.410 | 2.290 | 1.880 | 0.831 | 1.241 | 0.038 |
| Qafzeh | 17 | 0.972 | 0.321 | 0.900 | 0.941 | 0.282 | 0.550 | 1.860 | 1.310 | 1.182 | 1.061 | 0.078 |
| Skhul | 42 | 1.198 | 0.316 | 1.155 | 1.181 | 0.215 | 0.420 | 2.240 | 1.820 | 0.624 | 1.682 | 0.049 |

Table SI1.5.7: Results of multiple pairwise Wilcox tests for differences in scar proximal shape between sites.

|  | A5 | Al Wusta | BNS | MDF61 | Qafzeh |
| --- | --- | --- | --- | --- | --- |
| Al Wusta | 0.0204 | - | - | - | - |
| BNS | 0.0392 | 0.6995 | - | - | - |
| MDF61 | 0.0129 | 0.7888 | 0.5413 | - | - |
| Qafzeh | 0.0044 | 0.0583 | 0.1447 | 0.045 | - |
| Skhul | 4.30E+00 | 5 0.0204 | 0.1292 | 0.0087 | 0.7888 |

Table SI1.5.8: Results of multiple pairwise Wilcox tests for differences in scar proximal shape between regions.

|  | Eastern Africa | Arabia |
| --- | --- | --- |
| Arabia | 0.01321 | - |
| Levant | 2.60E-06 | 0.00026 |

Table SI1.5.9: Descriptive statistics of scar proximal shape by site.

|  | n | mean | sd | median | trimmed | mad | min | max | range | skew | kurtosis | se |
| --- | --- | --- | --- | --- | --- | --- | --- | --- | --- | --- | --- | --- |
| A5 | 79 | 1.106 | 0.265 | 1.070 | 1.080 | 0.208 | 0.750 | 2.400 | 1.650 | 1.772 | 5.803 | 0.030 |
| Al Wusta | 44 | 0.985 | 0.166 | 0.975 | 0.974 | 0.163 | 0.690 | 1.490 | 0.800 | 0.736 | 1.005 | 0.025 |
| BNS | 23 | 0.971 | 0.232 | 0.960 | 0.958 | 0.133 | 0.580 | 1.610 | 1.030 | 0.676 | 0.709 | 0.048 |
| MDF61 | 86 | 0.985 | 0.172 | 1.000 | 0.987 | 0.148 | 0.510 | 1.490 | 0.980 | 0.101 | 1.056 | 0.019 |
| Qafzeh | 17 | 0.838 | 0.245 | 0.790 | 0.835 | 0.252 | 0.480 | 1.240 | 0.760 | 0.288 | -1.483 | 0.060 |
| Skhul | 42 | 0.847 | 0.243 | 0.820 | 0.844 | 0.282 | 0.390 | 1.310 | 0.920 | 0.038 | -0.929 | 0.037 |

Table SI1.5.10: Results of multiple pairwise Wilcox tests for differences in scar distal shape between sites.

|  | A5 | Al Wusta | BNS | MDF61 | Qafzeh |
| --- | --- | --- | --- | --- | --- |
| Al Wusta | 0.164 | - | - | - | - |
| BNS | 0.164 | 0.694 | - | - | - |
| MDF61 | 0.016 | 0.409 | 0.997 | - | - |
| Qafzeh | 0.016 | 0.088 | 0.311 | 0.235 | - |
| Skhul | 0.997 | 0.26 | 0.26 | 0.067 | 0.048 |

Table SI1.5.11: Results of multiple pairwise Wilcox tests for differences in scar distal shape between regions.

|  | Eastern Africa | Arabia |
| --- | --- | --- |
| Arabia | 0.034 | - |
| Levant | 0.385 | 0.385 |

Table SI1.5.12: Descriptive statistics of scar distal shape by site.

|  | n | mean | sd | median | trimmed | mad | min | max | range | skew | kurtosis | se |
| --- | --- | --- | --- | --- | --- | --- | --- | --- | --- | --- | --- | --- |
| A5 | 79 | 1.720 | 0.982 | 1.350 | 1.551 | 0.445 | 0.630 | 6.040 | 5.410 | 1.946 | 4.062 | 0.111 |
| Al Wusta | 44 | 1.416 | 0.851 | 1.265 | 1.252 | 0.267 | 0.920 | 6.390 | 5.470 | 4.662 | 23.840 | 0.128 |
| BNS | 23 | 1.737 | 2.247 | 1.130 | 1.279 | 0.237 | 0.770 | 11.890 | 11.120 | 3.990 | 15.132 | 0.469 |
| MDF61 | 86 | 1.281 | 0.509 | 1.160 | 1.210 | 0.208 | 0.760 | 5.100 | 4.340 | 5.064 | 34.409 | 0.055 |
| Qafzeh | 17 | 1.119 | 0.198 | 1.100 | 1.117 | 0.208 | 0.810 | 1.450 | 0.640 | 0.250 | -1.374 | 0.048 |
| Skhul | 42 | 2.834 | 4.179 | 1.350 | 1.815 | 0.526 | 0.800 | 23.590 | 22.790 | 3.382 | 12.554 | 0.645 |

Table SI1.5.13: Scar termination type by site.

|  | Failed | Success |
| --- | --- | --- |
| A5 | 28 | 51 |
| Al Wusta | 11 | 33 |
| BNS | 12 | 11 |
| MDF61 | 27 | 59 |
| Qafzeh | 8 | 9 |
| Skhul | 7 | 38 |

Results of groupwise testing of termination type by site: X-squared = 13.164, df = 5, p-value = 0.02189

Results of groupwise testing of termination type by region: X-squared = 4.6454, df = 2, p-value = 0.09801

Table SI1.5.14: Results of multiple pairwise Wilcox tests for differences in scar to flaking face ratio between sites.

|  | A5 | Al Wusta | BNS | MDF61 | Qafzeh |
| --- | --- | --- | --- | --- | --- |
| Al Wusta | 0.8332 | - | - | - | - |
| BNS | 0.0828 | 0.0739 | - | - | - |
| MDF61 | 0.2874 | 0.2874 | 0.1818 | - | - |
| Qafzeh | 0.9847 | 0.8332 | 0.1818 | 0.7655 | - |
| Skhul | 0.0607 | 0.1194 | 0.0037 | 0.0113 | 0.1818 |

Table SI1.5.15: Results of multiple pairwise Wilcox tests for differences in scar to flaking face ratio between regions.

|  | Eastern Africa | Arabia |
| --- | --- | --- |
| Arabia | 0.899 | - |
| Levant | 0.016 | 0.016 |

Table SI1.5.16: Descriptive statistics of scar to flaking face ratio by site.

|  | n | mean | sd | median | trimmed | mad | min | max | range | skew | kurtosis | se |
| --- | --- | --- | --- | --- | --- | --- | --- | --- | --- | --- | --- | --- |
| A5 | 79 | 0.316 | 0.140 | 0.290 | 0.309 | 0.133 | 0.080 | 0.670 | 0.590 | 0.550 | -0.406 | 0.016 |
| Al Wusta | 44 | 0.335 | 0.167 | 0.310 | 0.314 | 0.141 | 0.120 | 0.850 | 0.730 | 1.143 | 0.849 | 0.025 |
| BNS | 23 | 0.238 | 0.131 | 0.220 | 0.231 | 0.163 | 0.040 | 0.520 | 0.480 | 0.412 | -1.019 | 0.027 |
| MDF61 | 86 | 0.311 | 0.216 | 0.255 | 0.284 | 0.126 | 0.090 | 1.790 | 1.700 | 3.830 | 22.911 | 0.023 |
| Qafzeh | 17 | 0.318 | 0.153 | 0.310 | 0.312 | 0.178 | 0.130 | 0.600 | 0.470 | 0.448 | -1.218 | 0.037 |
| Skhul | 42 | 0.402 | 0.178 | 0.390 | 0.390 | 0.208 | 0.150 | 0.860 | 0.710 | 0.509 | -0.417 | 0.027 |

**Supplementary Information 2: Results of Multivariate Analysis**

SI2.1: Preliminary Analysis

Table SI2.1.1: Key results of PCA for preliminary analysis reported in text.

|  |  | PC1 | PC2 |
| --- | --- | --- | --- |
| Eigenvalues | | 8.605691 | 2.614931 |
| Cumulative variation explained | | 41% | 53% |
| Variable Loadings | Weight | 0.30605 | 0.140303 |
|  | MaxDimension | 0.312818 | 0.036323 |
|  | AxialLength | 0.299338 | 0.096123 |
|  | ProxAxialWidth | 0.290974 | 0.132197 |
|  | MedAxialWidth | 0.325162 | -0.00731 |
|  | DistAxialWidth | 0.280997 | -0.19434 |
|  | MedThickness | 0.201659 | 0.224346 |
|  | Elongation | -0.08522 | 0.133877 |
|  | Flatness | 0.124709 | -0.23594 |
|  | DistShp | -0.00932 | 0.357854 |
|  | ProxShp | -0.04184 | 0.266295 |
|  | PlatformWidth | 0.286537 | 0.091396 |
|  | IPA | -0.01368 | 0.212585 |
|  | Scars_greater_than_5mm | 0.132397 | -0.1367 |
|  | DominantScarLength | 0.241407 | 0.226527 |
|  | DominantScarProxWidth | 0.273151 | 0.092905 |
|  | DominantScarMedWidth | 0.296111 | -0.06207 |
|  | DominantScarDistWidth | 0.232376 | -0.33686 |
|  | ScarElong | -0.06715 | 0.317696 |
|  | ScarProxShp | -0.07243 | 0.256525 |
|  | ScarDistShp | -0.00755 | 0.418711 |

Table SI2.1.2: Results of multiple pairwise Wilcox tests for differences in PC1 between sites.

|  | A5 | Al Wusta | BNS | MDF61 | Qafzeh |
| --- | --- | --- | --- | --- | --- |
| Al Wusta | 0.00152 | - | - | - | - |
| BNS | 0.03116 | 0.56779 | - | - | - |
| MDF61 | 1.60E-06 | 9.40E-12 | 1.30E-07 | - | - |
| Qafzeh | 0.00035 | 3.90E-07 | 8.10E-06 | 0.05165 | - |
| Skhul | 1.20E-08 | 8.70E-14 | 9.80E-10 | 0.00198 | 0.86569 |

Table SI2.1.3: Results of multiple pairwise Wilcox tests for differences in PC1 between regions.

|  | Eastern Africa | Arabia |
| --- | --- | --- |
| Arabia | 0.003 | - |
| Levant | 2.60E-12 | 2.10E-08 |

Table SI2.1.4: Descriptive statistics of PC1 by site.

|  | n | mean | sd | median | trimmed | mad | min | max | range | skew | kurtosis | se |
| --- | --- | --- | --- | --- | --- | --- | --- | --- | --- | --- | --- | --- |
| A5 | 78 | -0.91 | 2.76 | -1.00 | -0.87 | 2.28 | -7.44 | 5.99 | 13.42 | -0.13 | -0.06 | 0.31 |
| Al Wusta | 44 | -2.46 | 2.41 | -2.61 | -2.61 | 2.28 | -6.32 | 4.96 | 11.28 | 0.66 | 0.50 | 0.36 |
| BNS | 22 | -2.10 | 2.21 | -2.48 | -2.27 | 2.01 | -5.41 | 4.52 | 9.93 | 1.04 | 1.36 | 0.47 |
| MDF61 | 80 | 1.05 | 1.81 | 0.84 | 0.99 | 1.82 | -2.78 | 5.16 | 7.94 | 0.28 | -0.56 | 0.20 |
| Qafzeh | 17 | 2.62 | 3.26 | 2.20 | 2.59 | 2.35 | -2.42 | 8.16 | 10.58 | 0.28 | -0.98 | 0.79 |
| Skhul | 41 | 2.37 | 2.18 | 2.31 | 2.25 | 2.18 | -1.69 | 9.51 | 11.20 | 0.75 | 1.13 | 0.34 |

Table SI2.1.5: Results of multiple pairwise Wilcox tests for differences in PC2 between sites.

|  | A5 | Al Wusta | BNS | MDF61 | Qafzeh |
| --- | --- | --- | --- | --- | --- |
| Al Wusta | 0.004 | - | - | - | - |
| BNS | 0.0003 | 0.0581 | - | - | - |
| MDF61 | 3.60E-08 | 0.0906 | 0.1963 | - | - |
| Qafzeh | 2.50E-06 | 0.0028 | 0.2252 | 0.0057 | - |
| Skhul | 0.0073 | 0.8645 | 0.0835 | 0.1963 | 0.0053 |

Table SI2.1.6: Results of multiple pairwise Wilcox tests for differences in PC2 between regions.

|  | Eastern Africa | Arabia |
| --- | --- | --- |
| Arabia | 0.00011 | - |
| Levant | 0.00086 | 0.38203 |

Table SI2.1.7: Descriptive statistics of PC2 by site.

|  | n | mean | sd | median | trimmed | mad | min | max | range | skew | kurtosis | se |
| --- | --- | --- | --- | --- | --- | --- | --- | --- | --- | --- | --- | --- |
| A5 | 78 | 0.95 | 1.48 | 0.92 | 0.96 | 1.51 | -2.13 | 4.02 | 6.14 | -0.05 | -0.54 | 0.17 |
| Al Wusta | 44 | 0.07 | 1.60 | -0.05 | 0.01 | 1.42 | -3.32 | 5.37 | 8.69 | 0.64 | 1.28 | 0.24 |
| BNS | 22 | -0.83 | 1.60 | -1.35 | -0.90 | 1.44 | -3.41 | 2.43 | 5.84 | 0.42 | -0.81 | 0.34 |
| MDF61 | 80 | -0.47 | 1.20 | -0.36 | -0.47 | 1.13 | -3.89 | 4.26 | 8.16 | 0.32 | 2.19 | 0.13 |
| Qafzeh | 17 | -1.39 | 1.06 | -1.64 | -1.44 | 1.39 | -2.63 | 0.59 | 3.22 | 0.34 | -1.46 | 0.26 |
| Skhul | 41 | 0.04 | 1.82 | -0.16 | -0.04 | 1.76 | -3.23 | 4.36 | 7.59 | 0.38 | -0.51 | 0.28 |

SI2.2: Core Shape

Table SI2.2.1: Key results of PCA for preliminary analysis reported in text.

|  |  | PC1 | PC2 | PC3 | PC4 |
| --- | --- | --- | --- | --- | --- |
| Eigenvalues | | 1.388085 | 1.047346 | 0.888248 | 0.676322 |
| Cumulative variation explained | | 35% | 61% | 83% | 100% |
| Variable Loadings | Weight | 0.316722 | -0.65946 | 0.655016 | 0.18909 |
|  | Elongation | -0.67243 | -0.05649 | 0.055832 | 0.735885 |
|  | Flatness | 0.609096 | -0.05886 | -0.52463 | 0.591861 |
|  | DistShp | -0.27663 | -0.7473 | -0.54092 | -0.26911 |

Table SI2.2.2: Results of multiple pairwise Wilcox tests for differences in PC1 between sites.

|  | A5 | Al Wusta | BNS | MDF61 | Qafzeh |
| --- | --- | --- | --- | --- | --- |
| Al Wusta | 0.4862 | - | - | - | - |
| BNS | 0.1055 | 0.1157 | - | - | - |
| MDF61 | 1.80E-07 | 7.60E-05 | 0.0497 | - | - |
| Qafzeh | 5.10E-06 | 0.0003 | 0.0058 | 0.2119 | - |
| Skhul | 7.60E-07 | 0.0002 | 0.0433 | 0.967 | 0.1861 |

Table SI2.2.3: Results of multiple pairwise Wilcox tests for differences in PC1 between regions.

|  | Eastern Africa | Arabia |
| --- | --- | --- |
| Arabia | 0.00085 | - |
| Levant | 2.30E-09 | 0.0179 |

Table SI2.2.4: Descriptive statistics of PC1 by site.

|  | n | mean | sd | median | trimmed | mad | min | max | range | skew | kurtosis | se |
| --- | --- | --- | --- | --- | --- | --- | --- | --- | --- | --- | --- | --- |
| A5 | 78 | -0.48 | 0.92 | -0.47 | -0.51 | 0.74 | -3.08 | 3.28 | 6.36 | 0.58 | 2.59 | 0.10 |
| Al Wusta | 44 | -0.75 | 1.50 | -0.61 | -0.70 | 1.70 | -5.02 | 1.81 | 6.83 | -0.38 | -0.26 | 0.23 |
| BNS | 23 | -0.08 | 1.05 | -0.11 | -0.11 | 1.09 | -2.07 | 2.32 | 4.39 | 0.21 | -0.42 | 0.22 |
| MDF61 | 84 | 0.44 | 1.07 | 0.48 | 0.47 | 1.06 | -2.29 | 2.39 | 4.68 | -0.30 | -0.41 | 0.12 |
| Qafzeh | 17 | 0.81 | 0.73 | 0.78 | 0.82 | 0.63 | -0.81 | 2.15 | 2.96 | -0.08 | -0.30 | 0.18 |
| Skhul | 46 | 0.47 | 0.87 | 0.38 | 0.47 | 0.86 | -1.45 | 2.21 | 3.66 | -0.01 | -0.70 | 0.13 |

Table SI2.2.5: Descriptive statistics of PC1 by region.

|  | n | mean | sd | median | trimmed | mad | min | max | range | skew | kurtosis | se |
| --- | --- | --- | --- | --- | --- | --- | --- | --- | --- | --- | --- | --- |
| Eastern  Africa | 101 | -0.38 | 0.96 | -0.42 | -0.42 | 0.81 | -3.09 | 3.29 | 6.38 | 0.52 | 1.63 | 0.10 |
| Arabia | 128 | 0.03 | 1.35 | 0.29 | 0.11 | 1.24 | -5.06 | 2.39 | 7.45 | -0.68 | 0.50 | 0.12 |
| Levant | 63 | 0.56 | 0.85 | 0.54 | 0.56 | 0.83 | -1.47 | 2.21 | 3.68 | -0.09 | -0.52 | 0.11 |

Table SI2.2.6: Results of multiple pairwise Wilcox tests for differences in PC2 between sites.

|  | A5 | Al Wusta | BNS | MDF61 | Qafzeh |
| --- | --- | --- | --- | --- | --- |
| Al Wusta | 0.00031 | - | - | - | - |
| BNS | 0.024 | 0.31714 | - | - | - |
| MDF61 | 0.31714 | 0.00021 | 0.02552 | - | - |
| Qafzeh | 0.34224 | 0.10857 | 0.41607 | 0.41607 | - |
| Skhul | 0.16582 | 4.90E-06 | 0.00201 | 0.02552 | 0.05591 |

Table SI2.2.7: Results of multiple pairwise Wilcox tests for differences in PC2 between regions.

|  | Eastern Africa | Arabia |
| --- | --- | --- |
| Arabia | 0.0929 | - |
| Levant | 0.0929 | 0.0042 |

Table SI2.2.8: Descriptive statistics of PC2 by site.

|  | n | mean | sd | median | trimmed | mad | min | max | range | skew | kurtosis | se |
| --- | --- | --- | --- | --- | --- | --- | --- | --- | --- | --- | --- | --- |
| A5 | 78 | -0.12 | 1.11 | -0.33 | -0.18 | 0.74 | -2.52 | 3.02 | 5.54 | 0.63 | 0.57 | 0.13 |
| Al Wusta | 44 | 0.68 | 1.10 | 0.66 | 0.68 | 1.19 | -1.92 | 2.78 | 4.71 | -0.08 | -0.31 | 0.17 |
| BNS | 23 | 0.44 | 0.93 | 0.31 | 0.40 | 0.72 | -1.01 | 2.28 | 3.29 | 0.43 | -0.59 | 0.19 |
| MDF61 | 84 | -0.11 | 0.75 | 0.03 | -0.10 | 0.55 | -1.87 | 2.05 | 3.92 | -0.19 | -0.05 | 0.08 |
| Qafzeh | 17 | 0.17 | 0.91 | -0.09 | 0.13 | 0.94 | -0.97 | 1.80 | 2.76 | 0.32 | -1.39 | 0.22 |
| Skhul | 46 | -0.52 | 0.93 | -0.51 | -0.48 | 0.68 | -2.71 | 1.38 | 4.09 | -0.28 | -0.33 | 0.14 |

Table SI2.2.9: Descriptive statistics of PC2 by site.

|  | n | mean | sd | median | trimmed | mad | min | max | range | skew | kurtosis | se |
| --- | --- | --- | --- | --- | --- | --- | --- | --- | --- | --- | --- | --- |
| Eastern  Africa | 101 | 0.01 | 1.09 | -0.21 | -0.05 | 0.88 | -2.52 | 3.02 | 5.54 | 0.49 | 0.29 | 0.11 |
| Arabia | 128 | 0.16 | 0.96 | 0.13 | 0.13 | 0.77 | -1.92 | 2.78 | 4.71 | 0.32 | 0.30 | 0.08 |
| Levant | 63 | -0.33 | 0.97 | -0.40 | -0.31 | 0.80 | -2.71 | 1.80 | 4.50 | -0.12 | -0.18 | 0.12 |

**SI2.3: Flake Production**

Table SI2.3.1: Key results of PCA for preliminary analysis reported in text.

|  |  | PC1 | PC2 | PC3 | PC4 | PC5 | PC6 | PC7 | PC8 |
| --- | --- | --- | --- | --- | --- | --- | --- | --- | --- |
| Eigenvalues | | 1.84 | 1.37 | 1.08 | 1.02 | 0.83 | 0.79 | 0.57 | 0.50 |
| Cumulative variation explained | | 0.23 | 0.40 | 0.54 | 0.66 | 0.77 | 0.87 | 0.94 | 1.00 |
| Variable loadings | Weight | 0.16 | -0.57 | 0.22 | -0.36 | 0.48 | 0.01 | -0.44 | 0.22 |
|  | DistShp | -0.31 | -0.48 | 0.05 | 0.37 | -0.42 | -0.31 | -0.40 | -0.31 |
|  | IPA | -0.22 | -0.25 | -0.50 | -0.65 | -0.07 | -0.20 | 0.27 | -0.31 |
|  | Scars_greater_than_5mm | 0.40 | -0.48 | 0.16 | 0.25 | 0.08 | 0.28 | 0.53 | -0.40 |
|  | ScarElong | -0.44 | -0.08 | 0.10 | -0.17 | -0.24 | 0.83 | -0.11 | -0.02 |
|  | ScarProxShp | -0.35 | 0.05 | -0.42 | 0.39 | 0.68 | 0.12 | -0.11 | -0.25 |
|  | ScarDistShp | -0.53 | -0.27 | 0.18 | 0.14 | 0.10 | -0.19 | 0.51 | 0.54 |
|  | ScarFaceAreaRatio | -0.30 | 0.27 | 0.67 | -0.22 | 0.23 | -0.19 | 0.06 | -0.49 |

Table SI2.3.2: Results of multiple pairwise Wilcox tests for differences in PC1 between sites.

|  | A5 | Al Wusta | BNS | MDF61 | Qafzeh |
| --- | --- | --- | --- | --- | --- |
| Al Wusta | 0.13348 | - | - | - | - |
| BNS | 0.00226 | 0.02852 | - | - | - |
| MDF61 | 2.00E-06 | 0.00705 | 0.35178 | - | - |
| Qafzeh | 1.10E-06 | 4.10E-06 | 0.02852 | 0.00012 | - |
| Skhul | 0.00096 | 0.02852 | 0.57959 | 0.71245 | 0.00148 |

Table SI2.3.3: Results of multiple pairwise Wilcox tests for differences in PC1 between regions.

|  | Eastern Africa | Arabia |
| --- | --- | --- |
| Arabia | 0.0023 | - |
| Levant | 2.80E-05 | 0.0022 |

Table SI2.3.4: Descriptive statistics of PC1 by site.

|  | n | mean | sd | median | trimmed | mad | min | max | range | skew | kurtosis | se |
| --- | --- | --- | --- | --- | --- | --- | --- | --- | --- | --- | --- | --- |
| A5 | 78 | -0.71 | 1.24 | -0.61 | -0.75 | 1.20 | -3.02 | 2.78 | 5.80 | 0.22 | -0.32 | 0.14 |
| Al Wusta | 44 | -0.35 | 1.22 | -0.28 | -0.35 | 1.03 | -3.11 | 3.08 | 6.19 | 0.02 | 0.31 | 0.18 |
| BNS | 23 | 0.48 | 1.65 | 0.86 | 0.54 | 1.85 | -3.14 | 3.08 | 6.22 | -0.42 | -0.75 | 0.34 |
| MDF61 | 81 | 0.26 | 0.97 | 0.26 | 0.27 | 0.94 | -2.98 | 2.58 | 5.56 | -0.23 | 0.58 | 0.11 |
| Qafzeh | 17 | 1.66 | 1.09 | 1.96 | 1.71 | 1.15 | -0.39 | 3.00 | 3.39 | -0.53 | -1.19 | 0.26 |
| Skhul | 41 | 0.25 | 1.43 | 0.48 | 0.28 | 1.34 | -2.34 | 2.54 | 4.88 | -0.27 | -1.04 | 0.22 |

Table SI2.3.5: Descriptive statistics of PC1 by region.

|  | n | mean | sd | median | trimmed | mad | min | max | range | skew | kurtosis | se |
| --- | --- | --- | --- | --- | --- | --- | --- | --- | --- | --- | --- | --- |
| Eastern  Africa | 101 | -0.44 | 1.42 | -0.45 | -0.48 | 1.42 | -3.14 | 3.08 | 6.22 | 0.25 | -0.47 | 0.14 |
| Arabia | 125 | 0.05 | 1.10 | 0.01 | 0.07 | 0.93 | -3.11 | 3.08 | 6.19 | -0.27 | 0.53 | 0.10 |
| Levant | 58 | 0.66 | 1.48 | 0.71 | 0.73 | 1.64 | -2.34 | 3.00 | 5.34 | -0.40 | -0.84 | 0.19 |

Table SI2.3.6: Results of multiple pairwise Wilcox tests for differences in PC2 between sites.

|  | A5 | Al Wusta | BNS | MDF61 | Qafzeh |
| --- | --- | --- | --- | --- | --- |
| Al Wusta | 0.0046 | - | - | - | - |
| BNS | 0.6167 | 0.1374 | - | - | - |
| MDF61 | 0.6423 | 0.0046 | 0.6423 | - | - |
| Qafzeh | 0.7903 | 0.0508 | 0.7225 | 0.985 | - |
| Skhul | 0.0508 | 2.10E-05 | 0.0508 | 0.0078 | 0.068 |

Table SI2.3.7: Results of multiple pairwise Wilcox tests for differences in PC2 between regions.

|  | Eastern Africa | Arabia |
| --- | --- | --- |
| Arabia | 0.07707 | - |
| Levant | 0.07208 | 0.00062 |

Table SI2.3.8: Descriptive statistics of PC2 by site.

|  | n | mean | sd | median | trimmed | mad | min | max | range | skew | kurtosis | se |
| --- | --- | --- | --- | --- | --- | --- | --- | --- | --- | --- | --- | --- |
| A5 | 78 | -0.06 | 1.28 | -0.15 | -0.12 | 0.89 | -3.32 | 3.93 | 7.25 | 0.50 | 0.87 | 0.15 |
| Al Wusta | 44 | 0.70 | 1.30 | 0.63 | 0.69 | 1.28 | -2.90 | 3.36 | 6.26 | -0.22 | 0.40 | 0.20 |
| BNS | 23 | 0.04 | 1.26 | 0.26 | 0.09 | 1.07 | -2.77 | 2.10 | 4.87 | -0.47 | -0.53 | 0.26 |
| MDF61 | 81 | -0.03 | 0.89 | 0.02 | -0.01 | 0.84 | -2.28 | 2.61 | 4.89 | -0.12 | 0.23 | 0.10 |
| Qafzeh | 17 | -0.01 | 0.88 | 0.03 | -0.03 | 1.15 | -1.24 | 1.61 | 2.85 | 0.22 | -1.35 | 0.21 |
| Skhul | 41 | -0.59 | 1.02 | -0.61 | -0.62 | 0.99 | -2.59 | 1.61 | 4.20 | 0.22 | -0.55 | 0.16 |

Table SI2.3.9: Descriptive statistics of PC2 by region.

|  | n | mean | sd | median | trimmed | mad | min | max | range | skew | kurtosis | se |
| --- | --- | --- | --- | --- | --- | --- | --- | --- | --- | --- | --- | --- |
| Eastern Africa | 101 | -0.04 | 1.27 | -0.02 | -0.07 | 1.03 | -3.32 | 3.93 | 7.25 | 0.29 | 0.59 | 0.13 |
| Arabia | 125 | 0.22 | 1.10 | 0.15 | 0.20 | 0.96 | -2.90 | 3.36 | 6.26 | 0.18 | 0.68 | 0.10 |
| Levant | 58 | -0.42 | 1.01 | -0.42 | -0.43 | 1.05 | -2.59 | 1.61 | 4.21 | 0.12 | -0.62 | 0.13 |

Table SI2.3.10: Results of multiple pairwise Wilcox tests for differences in PC3 between sites.

|  | A5 | Al Wusta | BNS | MDF61 | Qafzeh |
| --- | --- | --- | --- | --- | --- |
| Al Wusta | 0.1619 | - | - | - | - |
| BNS | 0.4915 | 0.7481 | - | - | - |
| MDF61 | 0.4915 | 0.0509 | 0.2531 | - | - |
| Qafzeh | 0.01 | 0.0019 | 0.0124 | 0.0314 | - |
| Skhul | 2.10E-07 | 1.60E-08 | 4.60E-06 | 2.40E-06 | 0.4386 |

Table SI2.3.11 Results of multiple pairwise Wilcox tests for differences in PC3 between regions.

|  | Eastern Africa | Arabia |
| --- | --- | --- |
| Arabia | 0.91 | - |
| Levant | 1.40E-09 | 1.40E-09 |

Table SI2.3.12: Descriptive statistics of PC3 by site.

|  | n | mean | sd | median | trimmed | mad | min | max | range | skew | kurtosis | se |
| --- | --- | --- | --- | --- | --- | --- | --- | --- | --- | --- | --- | --- |
| A5 | 78 | -0.20 | 0.83 | -0.30 | -0.19 | 0.85 | -1.81 | 1.52 | 3.33 | 0.08 | -0.81 | 0.09 |
| Al Wusta | 44 | -0.46 | 0.84 | -0.56 | -0.47 | 0.94 | -2.19 | 1.45 | 3.64 | 0.10 | -0.62 | 0.13 |
| BNS | 23 | -0.44 | 1.07 | -0.45 | -0.40 | 0.79 | -2.99 | 1.76 | 4.75 | -0.40 | 0.37 | 0.22 |
| MDF61 | 81 | -0.07 | 0.91 | -0.07 | -0.11 | 0.85 | -1.62 | 2.80 | 4.43 | 0.46 | 0.13 | 0.10 |
| Qafzeh | 17 | 0.67 | 1.14 | 0.82 | 0.68 | 1.60 | -1.31 | 2.47 | 3.78 | 0.01 | -1.33 | 0.28 |
| Skhul | 41 | 0.99 | 1.04 | 0.94 | 0.96 | 1.21 | -1.69 | 3.50 | 5.19 | 0.06 | -0.17 | 0.16 |

Table SI2.3.13: Descriptive statistics of PC3 by region.

|  | n | mean | sd | median | trimmed | mad | min | max | range | skew | kurtosis | se |
| --- | --- | --- | --- | --- | --- | --- | --- | --- | --- | --- | --- | --- |
| Eastern Africa | 101 | -0.25 | 0.89 | -0.30 | -0.24 | 0.85 | -2.99 | 1.76 | 4.75 | -0.18 | 0.10 | 0.09 |
| Arabia | 125 | -0.21 | 0.90 | -0.22 | -0.24 | 0.90 | -2.19 | 2.80 | 4.99 | 0.37 | 0.11 | 0.08 |
| Levant | 58 | 0.89 | 1.07 | 0.83 | 0.89 | 1.30 | -1.69 | 3.50 | 5.19 | 0.01 | -0.46 | 0.14 |

**SI2.4: Multiple Matrix Regressions**

Table SI2.4.1: Results of multiple matrix regressions of core shaping with 50km radius spatial variables.

| **Multiple R-squared** | | | 0.023384 |
| --- | --- | --- | --- |
| **F-Statistic** | | | 191.0569 |
| **p value (9999 permutations)** | | | 2.00E-04 |
| **Coefficients:** | **Estimate** | **t value** | **Pr(>\|t\|)** |
| **(intercept)** | 0.284632 | 255.6645 | 1 |
| **Cost path** | 0.023575 | 9.577897 | 0.0001 |
| **Altitude** | 0.001813 | 0.424985 | 0.938394 |
| **Terrain Roughness** | 0.033853 | 4.483764 | 0.512051 |
| **Temperature** | 0.041858 | 8.749182 | 0.124212 |
| **Precipitation** | -0.07357 | -9.16113 | 0.144914 |

Table SI2.4.2: Results of multiple matrix regressions of core shaping with 5km radius spatial variables.

| **Multiple R-squared** | | | 0.02246 |
| --- | --- | --- | --- |
| **F-Statistic** | | | 183.3378 |
| **p value (9999 permutations)** | | | 2.00E-04 |
| **Coefficients:** | **Estimate** | **t value** | **Pr(>\|t\|)** |
| **(intercept)** | 0.282748 | 254.1315 | 1 |
| **Cost path** | 0.023899 | 10.74426 | 0.0001 |
| **Altitude** | 0.010496 | 2.862782 | 0.555356 |
| **Terrain Roughness** | 0.00811 | 2.69682 | 0.643864 |
| **Temperature** | 0.029798 | 9.779344 | 0.057106 |
| **Precipitation** | -0.05247 | -11.4483 | 0.0004 |

Table SI2.4.3: Results of multiple matrix regressions of flake production with 50km radius spatial variables.

| **Multiple R-squared** | | | 0.054169 |
| --- | --- | --- | --- |
| **F-Statistic** | | | 456.9913 |
| **p value (9999 permutations)** | | | 1.00E-04 |
| **Coefficients:** | **Estimate** | **t value** | **Pr(>\|t\|)** |
| **(intercept)** | 0.36518 | 344.0009 | 1 |
| **Cost path** | 0.034187 | 14.56595 | 0.0001 |
| **Altitude** | 0.130501 | 32.0774 | 0.0001 |
| **Terrain Roughness** | -0.14249 | -19.7929 | 0.005701 |
| **Temperature** | -0.14022 | -30.7373 | 0.0001 |
| **Precipitation** | 0.144831 | 18.91471 | 0.005001 |

Table SI2.4.4: Results of multiple matrix regressions of core shaping with 5km radius spatial variables.

| **Multiple R-squared** | | | 0.055535 |
| --- | --- | --- | --- |
| **F-Statistic** | | | 469.188223, |
| **p value (9999 permutations)** | | | 1.00E-04 |
| **Coefficients:** | **Estimate** | **t value** | **Pr(>\|t\|)** |
| **(intercept)** | 0.365037 | 344.4926 | 1 |
| **Cost path** | 0.031071 | 14.66662 | 0.0001 |
| **Altitude** | 0.120183 | 34.4181 | 0.0001 |
| **Terrain Roughness** | -0.0068 | -2.37542 | 0.689269 |
| **Temperature** | -0.09004 | -31.0281 | 0.0001 |
| **Precipitation** | 0.031934 | 7.315335 | 0.026503 |
